# Cyclic Compression Testing of Three Elastomer Types—A Thermoplastic Vulcanizate Elastomer, a Liquid Silicone Rubber and Two Ethylene-Propylene-Diene Rubbers

**DOI:** 10.3390/polym14071316

**Published:** 2022-03-24

**Authors:** Anna-Maria Märta Ruth Persson, Erik Andreassen

**Affiliations:** 1 Dept. of Manufacturing and Civil Engineering, NTNU, P.O. Box 191, 2802 Gjøvik, Norway; 2 Polymer and Composite Materials Group, SINTEF Industry, P.O. Box 124 Blindern, 0314 Oslo, Norway

**Keywords:** hysteresis, Mullins effect, compression set, Poisson’s ratio, stress relaxation, strain recovery

## Abstract

Thermoplastic elastomer vulcanizate (TPV) and liquid silicone rubber (LSR) are replacement candidates for ethylene-propylene-diene rubbers (EPDM), as they offer the possibility for two-component injection moulding. In this study, these material types were compared side by side in cyclic compression tests. The materials were also characterized to provide details on the formulations. Compared to the rubbers, the TPV had higher compression set (after a given cycle) and hysteresis loss, and a stronger Mullins effect. This is due to the thermoplastic matrix in the TPV. The LSR had lower compression set (after a given cycle) than the EPDM, but stronger Mullins effect and higher relative hysteresis loss. These differences between the LSR and the EPDM are likely due to differences in polymer network structure and type of filler. Methods for quantifying the Mullins effect are proposed, and correlations between a Mullins index and parameters such as compression set are discussed. The EPDMs showed a distinct trend in compression set, relative hysteresis loss and relaxed stress fraction vs. strain amplitude; these entities were almost independent of strain amplitude in the range 15–35%, while they increased in this range for the TPV and the LSR. The difference between the compression set values of the LSR and the EPDM decreased with increasing strain amplitude and increasing strain recovery time.

## 1. Introduction

The purpose of this article is to compare three types of elastomer materials with regard to their behaviour in cyclic compression, including properties/phenomena such as the Mullins effect, compression set and hysteresis loss. The materials in this study are a thermoplastic vulcanizate elastomer (TPV), a liquid silicone rubber (LSR) and two ethylene-propylene-diene M class rubbers (EPDM). These three elastomer types have somewhat different properties, but they may all be used as seals and gaskets in combination with (hard) plastic materials; EPDM in the form of mounted parts (e.g., O-rings), and the chosen TPV and LSR as over-moulded seals and gaskets via two-component injection moulding. Hence, TPVs and LSRs may replace EPDMs in certain applications, by utilizing the process-integrated assembly offered by two-component injection moulding, to make parts consisting of hard and soft polymeric materials [1]. The TPV in this study has been the subject of two other studies by the authors; one study of the temperature dependence of the compression behaviour [2], and one on the TPV’s adhesion to hard polyamide materials in two-component injection moulding [3,4].

Elastomers (rubbers and thermoplastic elastomers) are soft materials which are highly elastic up to large strains [5,6]. A rubber consists of a network of polymer chains crosslinked by covalent chemical bonds. The chains are based on one or a few different repeating units. Rubber compounds are crosslinked at elevated temperature, followed by post-curing and degassing at elevated temperature [5,6]. Thermoplastic elastomers (TPEs) have been commercially available for nearly 40 years, and offer elastomeric response in combination with thermoplastic processing, e.g., injection moulding [6,7]. In the TPE category, TPVs typically have the best properties in terms of rubber-like elasticity [7]. A TPV is a dynamically vulcanized blend of a thermoplastic polymer and a (dispersed) rubber [7]. The performance of a TPV is typically inferior to that of a rubber, e.g., with regard to properties such as compression set [7]. However, the performance of TPVs has been continuously improved and these materials are performing well in many situations [7].

Liquid silicone rubber (LSR) can be injection moulded with specialized equipment and cures (crosslinks) in a heated mould [5,8]. The most common LSRs are based on vinyl methyl silicone pre-polymers, but there are also other chemistries (with the same siloxane backbone) for certain applications. In addition to the pre-polymer, the uncured LSR compound contains crosslinker, catalyst and filler [5]. The uncured compound typically consists of two parts; one with the crosslinker and one with the catalyst. For LSR, the crosslinking reaction is a platinum-catalysed hydrosilylation reaction between Si-H on a (short) crosslinker and a vinyl end group on a (longer) pre-polymer [5]. LSRs are sometimes post-cured [6,8]. The post-curing can serve a dual purpose: to finalize the crosslinking (if uncomplete) and to remove volatiles such as short chain siloxanes [9].

The response of rubbers to cyclic loading has been studied since the 1940s [10], and due to the complexity of the mechanisms it remains a topic for the scientific community [11]. Characteristic features of the response to cyclic loading include the Mullins effect [10,11,12], and an evolution of the stress-strain envelope, including hysteresis loss and an increase in the residual strain after unloading. Some of these effects are also present in hard thermoplastics [13].

The properties of an elastomer depend on three main factors:

1. The polymer chain network: The properties of the network depend on the distribution of chain segment lengths between crosslinks, and the type of crosslink (which affects flexibility and strength). Shorter segments between crosslinks give smaller hysteresis loss and lower compression set. The properties of the network are also affected by its chain entanglements, dangling chains (with one end free), chain loops (with both ends connected to the same crosslink) and unconnected free chains. Note that the network properties, such as crosslink density, may vary within a thick part, which calls for special attention to the curing agents and processing parameters [14,15].
2. The interaction between the polymer matrix and the filler particles (e.g., carbon black particles in rubber or rubber particles in TPVs): The interphase between matrix and particles affects the elastomer performance [16,17]. Adding carbon black to a rubber will increase the stiffness, hardness and strength, but it will generally also give a stronger Mullins effect and larger hysteresis [5,17,18]. The interaction between rubber and carbon black particles is partly physical (adsorption) so that an increased surface area of the particles will reduce the Mullins effect and the hysteresis loss [5,17]. Filler particles act as an additional type of crosslink in the network, as they offer connections between polymer chains [17]. Compatibilization between the TPV phases improves the tensile properties, and also reduces the size of the rubber particles [19].
3. The filler properties and filler-filler interactions, i.e., surface area, size distribution, dispersion, volume fraction, etc.: Filler particles may agglomerate, and need to be broken up to achieve good dispersion. Silica particles are harder to disperse than carbon black particles, due to the stronger interaction between silica particles (hydrogen bonds) than between carbon black particles (van der Waals) [5,6,20].

For TPVs, the elastomeric character is enhanced with an increased rubber fraction [21]. However, the thermoplastic fraction needs to be above a certain minimum threshold in order to enable the material to be processable. Babu et al. [21] studied the cyclic tensile loading of PP-based TPVs with EPDM and other rubber phases. They observed that the thermoplastic phase contributed with stiffness to the TPV at the expense of increased hysteresis loss and residual strain [21]. Similar trends were reported by Liu et al. [22] for a TPV of HDPE with EPDM as the rubber phase. Regarding recoverability of TPVs after compressive cyclic testing, Wang et al. [23] reported that most of the residual strain and parts of the original stress-strain curve could be recovered with heat treatment [23].

For silicone rubbers, the type of curing method affects the properties. In a comparison between LSR (addition cured with platinum catalyst) and high consistency (silicone) rubber (cured with peroxide which is a radical curing), the LSR had lower hysteresis loss and lower residual strain [24]. This was explained by a more regular network in the LSR, with a narrower distribution of molar mass between crosslinks and fewer dangling chains [24] (controlled by the molar mass distribution of the pre-polymer [25]. Ref. [25] studied the relation between elastomer properties and network structure, and reported that a broader distribution of molar mass between crosslinks may improve the tear resistance. Ref. [26] analysed commercial LSR pre-polymers, which all had bimodal molar mass distributions, and the properties of the resulting networks. Ref. [27] studied the effect of crosslinker concentration (three levels) for an LSR. The crosslinker concentration affected the crosslink density and the concentration of dangling chains [27]. In monotonic compression tests, the lowest crosslinker concentration gave a lower modulus, but a higher strength [27]. In cyclic compressive tests, the crosslinker concentration had distinct effects on the evolution of peak stress, hysteresis loss and residual strain [27].

Hanson et al. [12] performed experiments to understand the mechanism of the Mullins effect in a peroxide-cured silicone rubber with silica filler. The rubber was subjected to cyclic tensile testing with a stepwise increase in strain (from 100 to 300%). A Mullins effect was observed, but not if the second loading was performed in a direction orthogonal to first load direction. Based on this and other observations, the authors proposed a mechanism for the Mullins effect, in which the higher tensile stress in the first loading, compared to the second, is due to chain entanglements being removed near the chains’ attachment points to filler particles. Clément et al. [28] also studied the Mullins effect in a peroxide-cured silicone rubber with silica filler. They attributed the effect to chains which had reached their limit of extensibility, and detached from, or slipped on, particle surfaces, mainly in regions with high local filler concentration.

Candau et al. [16] studied damage mechanisms in EPDM rubber vs. filler content in cyclic tensile tests. For medium filler content (<40 phr), the main damage mechanism was an irreversible damage to the network (rupture of chains and/or crosslinks). For high filler content (>40 phr), there was a transition, when the strain exceeded a certain value, to a damage in the filler network, involving creation of voids adjacent to the fillers, and cyclic loading favoured closing of voids upon unloading. These explanations were based on observed trends for the volumetric strain during cyclic tensile loading.

Litvinov et al. [17] used NMR to show that EPDM chain segments were strongly immobilized on the surface of carbon black particles. Based on a study with unfilled and carbon-black-filled EPDM rubbers in cyclic tensile tests, as well as modelling, ref. [17] concluded the following regarding the role of the physical junctions formed by EPDM chain segments absorbed at filler surfaces: A relatively small amount of strongly adsorbed chain segments can have a significant effect on the stress-strain response. This physical network may help in redistribution of local strains via slippage of the physical junctions along the carbon black surface. Furthermore, bridging chains increase the energy required for the breakdown of filler aggregates, and provide a source for energy dissipation via filler aggregate breakdown and reaggregation during loading and unloading. Their model reproduced filler-induced effects on cyclic tensile stress-strain curves, i.e., the stiffening effect, the Mullins effect and hysteresis.

Ehrburger-Dolle et al. [29,30] studied the effect of filler-matrix interaction strength vs. filler-filler interaction strength, using EPDM materials with three types of fillers: carbon black (strong matrix-filler interaction), hydrophobic silica and hydrophilic silica (the latter with the strongest filler-filler interaction). Tensile stress relaxation experiments were combined with X-ray photon correlation spectroscopy which probed the relaxation of the filler particles. For systems with stronger filler-matrix interactions, they observed reduced relaxation, hysteresis loss and Mullins effect.

The cyclic stress-strain response of TPEs, EPDM rubbers and LSRs has been the topic of many studies, but few have studied them side by side. Refs. [22,23] compared TPVs with rubbers, but the materials had quite different hardnesses because the rubbers were also constituents in the respective TPVs.

This study is based on cyclic compression tests of the three elastomer types mentioned above, using materials with similar hardness. The aim is to identify differences and similarities between these elastomer types, regarding key properties such as the Mullins effect, compression set and hysteresis loss.

## 2. Materials and Methods

### 2.1. Materials and Specimen Preparation

One thermoplastic elastomer and three rubbers (Table 1) were analysed in this study. The three first materials in the table have similar hardness values, while number three and four are two EPDMs with different hardness values.

The TPV is intended for two-component injection moulding and it is modified for adhesion to polyamides. Note that similar TPVs from the same manufacturer, but without this modification, have better compression set than the TPV in this study. The weight fraction of PP was estimated to be ~24% [2].

The LSR may be used in soft-hard two-component injection moulding (with special injection units for LSR), in combination with thermoplastic materials, and it is specified to have good adhesion to polyamides. The two EPDMs are not available as raw materials, but in the form of gaskets and seals, such as O-rings.

The LSR was received in the form of 2.5 mm thick sheets, crosslinked (catalysed by platinum) in a press at 165 °C for 5 min. (This LSR can also be post-cured, but the material in this study was not post-cured.) The EPDMs were received from the manufacturer in the form of 2 mm and 6 mm thick sheets, peroxide crosslinked in a press at 170 °C for 15 min, and post-cured for 3 h at 150 °C. The TPV was injection moulded in the authors’ lab as 80 mm × 80 mm plates with thickness 3.4 mm. The moulding parameters were chosen within the supplier’s recommendations, and the injection rate was set so that the flow front speed was the same as specified in ISO 294-1 for type 1A tensile specimens of ISO 527-2.

### 2.2. Thermal Characterization

Since the materials in the study are commercial grades, with limited information on the compositions, the materials were subjected to a thermal analysis.

Differential scanning calorimetry (DSC) was performed with a Discovery DSC 2500 (TA Instruments, New Castle, DE, USA). The heating/cooling rate was 20 °C/min. Heating and cooling in the interval −70 °C to 200 °C were performed twice.

Thermogravimetric analysis (TGA) was performed with a Discovery TGA 550 (TA Instruments), following ISO 9924-3 with minor adjustments: The carbon-based elastomers (TPV and EPDMs) where heated in nitrogen to 600 °C and cooled to 400 °C, before switching to air and heating to 900 °C. The isothermal holding times at 600 °C, 400 °C and 900 °C were 2 min, 2 min and 30 min, respectively. The LSR was analysed in a similar way, but it was heated to 800 °C in the first step.

### 2.3. Mechanical Testing

#### 2.3.1. Description of Tests and Procedures

Three types of cyclic compression tests were performed (Table 2), in order to investigate effects of cycle number and strain amplitude, as well as time effects (stress relaxation and strain recovery). In addition to these compression tests, three of the materials were also tested in tension.

The cylindrical specimens for compressive testing were punched from the sheets or plates with a rotating die lubricated with a soap-in-water solution. Note that the TPV specimens were compressed in the thickness direction of the injection-moulded plate. The compression tests were performed with a universal testing machine (Z250, ZwickRoell, Ulm, Germany) at 23 °C. Loading and unloading were performed with a constant crosshead speed (around 5 mm/min), selected so that the initial strain rate during loading was the same for all materials (0.013 s^−1^, as in the standard ISO 7743), despite somewhat different initial specimen heights.

If not otherwise stated, reported strains and stresses are engineering strains and stresses (the strain amplitude is based on the displacement of the crosshead and the initial specimen height). The pre-force was 2 N. The specimens were lubricated with silicone-based grease (Molykote PG 54, Dow Corning, Midland, MI, USA) to minimize the friction towards the steel compression plates.

#### 2.3.2. Optical Measurements of Radial Displacement

**Figure 1:**
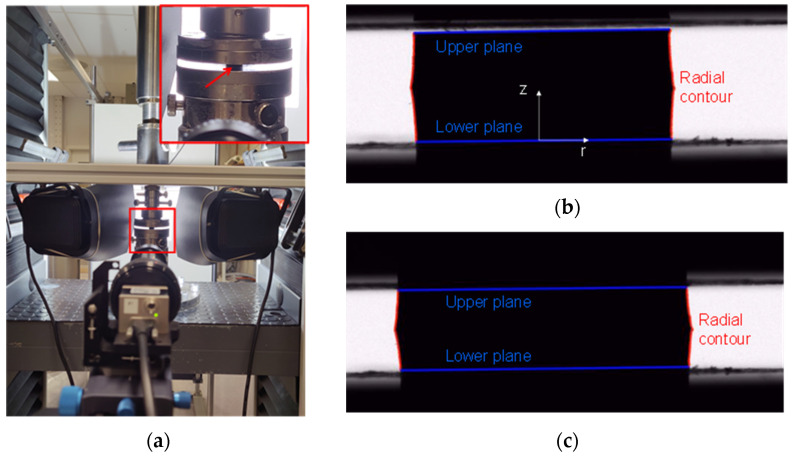
Measurement of radial displacements during compression: (**a**) experimental setup. The inset is a magnified view of the compression plates, and the red arrow points to the specimen; (**b**,**c**) are photos indicating the radial contour before and during testing, respectively.

A single camera (Basler acA4112-20um with a Rodagon 135 lens and a 48 mm extension tube) was aligned in front of the specimen, which was back lit, and focused on the radial contour (Figure 1a). The images were analysed with a MATLAB script, which calculated the horizontal gradient of the greytones. The radial contours were identified where the greytone turned from either white to black or vice versa (marked red by the script, see Figure 1b). An average radial strain (ε_r_) was calculated from the average of the radial displacements (Figure 1b,c).

The Poisson’s ratio, *ν*, was calculated from the radial and axial Hencky strains (*ε_r_* and *ε_z_*); see Appendix A.

(1) $$\nu =-\varepsilon_{r}/\varepsilon_{z}$$

#### 2.3.3. Parameters Derived from the Compression Tests

Parameters derived from the stress-strain data are listed in Table 3. These were obtained with a script which fitted polynomial functions to the individual loading and unloading curves; see Appendix B.1 for details. The parameters in Table 3 allow for comparison between materials and cycles. *TM*, *CS* and *HL* are key mechanical characteristics of elastomers. Note that *CS* in this study is different from a standard compression set (ISO 815-1:2019), which is the permanent set after a long compression duration. In this study, the test duration is short and *CS* is an instant value after unloading, without waiting for complete strain recovery. For the parameter *CS_R_* the recovery is nearly complete, but the degree of recovery differs between the materials. The Mullins indices *MI*_0_–*MI*_2_ are proposed in this study to quantify the Mullins effect (see Appendix B.2 for details). The parameters *ϕ_σ_* and *ϕ_ε_* describe the stress relaxation and strain recovery.

## 3. Results

### 3.1. Thermal Characterization

**Figure 2:**
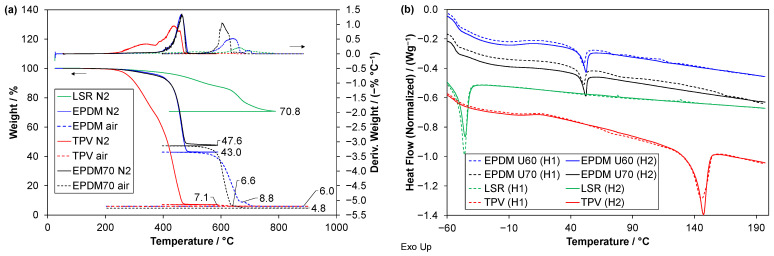
(**a**) TGA of the four materials; relative weight versus temperature (left axis) and derived weight (right axis). Solid lines and dashed lines indicate N_2_ and air atmosphere, respectively. (**b**) DSC of the four materials for first and second heating (denoted H1 and H2). The curves in (**b**) are shifted vertically for clarity.

TGA (Figure 2a) allows for determining the weight fractions of the constituents of the EPDMs and the TPV; see Table 4. Although LSRs are typically filled with up to about 30% silica particles, the silica content cannot be determined by TGA without analysing the parts A and B separately, due to reactions between the silica particles and the matrix [24,25].

The thermal stability of the materials can be estimated from the temperature at which the weight is reduced to 95% (T_95%_ in Table 4). The TPV is less thermally stable than the rubbers, while the LSR is more stable than the EPDMs. The DSC thermograms (Figure 2b) allow for identification of glass transition temperatures (T_g_) and melting temperatures (T_m_); see Table 4.

### 3.2. Cyclic Compression Tests

#### 3.2.1. Main Characteristics of the Stress-Strain Curves

**Figure 3:**
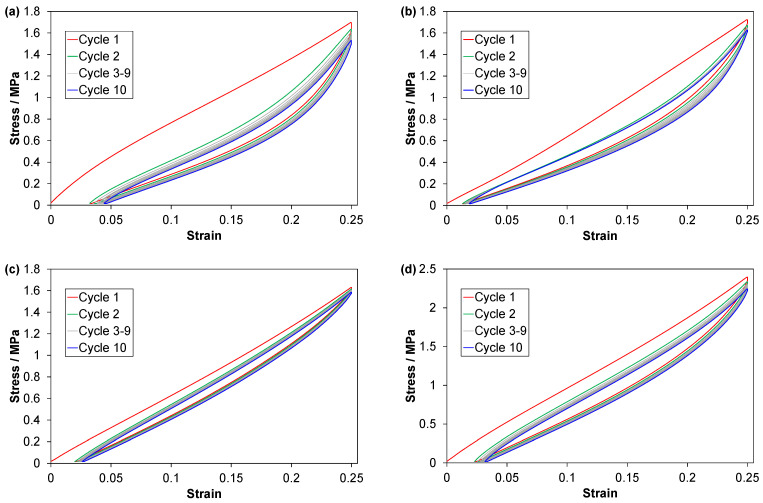
Data from C1 tests for (**a**) TPV, (**b**) LSR, (**c**) EPDM1 and (**d**) EPDM2. Data for one representative test of each material. Note the different ordinate scale for EPDM2 in (**d**).

Stress-strain curves from C1 tests are shown in Figure 3. The TPV experienced a marked softening during the first loading, up to a strain of about 0.05 (Figure 3a). The derivatives (Appendix C, Figure A3) show a weak softening of the EPDMs, and a weak hardening of the LSR.

The effect of cycling on the loading-unloading loops can be described as a combination of a horizontal (strain) shift and a vertical shift. The vertical shift mainly affects the highest stresses. For the TPV and the EPDMs, the horizontal shifts are quite uniform for all strains, while they are non-uniform for the LSR. The TPV shows the strongest reduction in peak stress and the largest increase in residual strain upon cycling. For the LSR, the horizontal shift of the loading curves is nearly zero at low strains and the shift is largest for the unloading curves at intermediate to high strains (Figure 3b). If the loops of cycle 2–10 are arranged so that all have a common origin (i.e., a common starting point for the loading curves), it follows that the LSR shows a weak initial hardening upon cycling (see also derivatives for 2nd and 10th loading in Appendix C, Figure A3).

**Figure 4:**
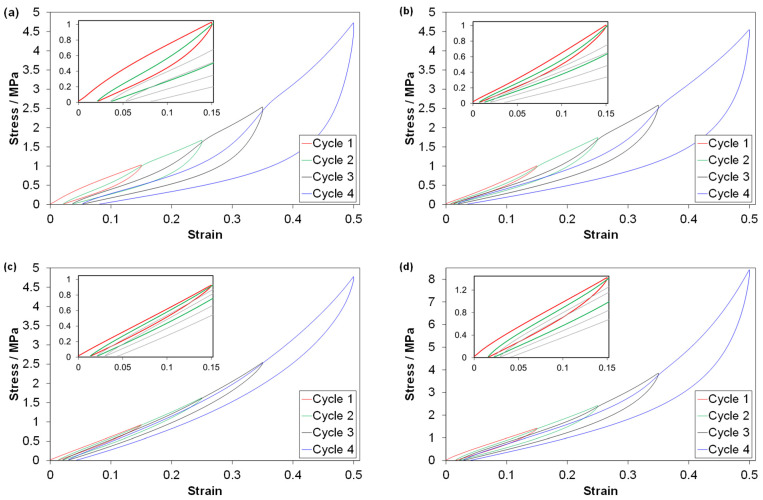
Data from C2 tests for (**a**) TPV, (**b**) LSR, (**c**) EPDM1 and (**d**) EPDM2. Data for one representative test of each material. Note the different ordinate scale for (**d**). The insets are magnified views of the first cycle.

Stress-strain curves from C2 tests are shown in Figure 4. The reloading curves of the TPV and the LSR show a clear Mullins (softening) effect (best seen for the last reloading in this figure), while this effect is weak for the EPDMs.

**Figure 5:**
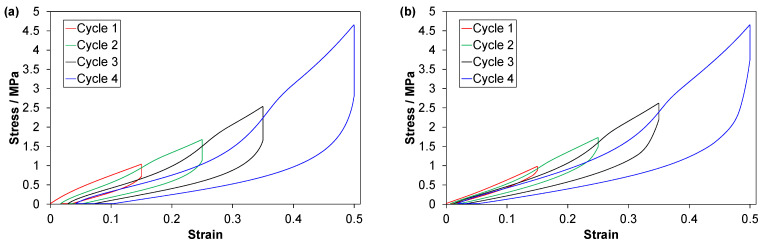

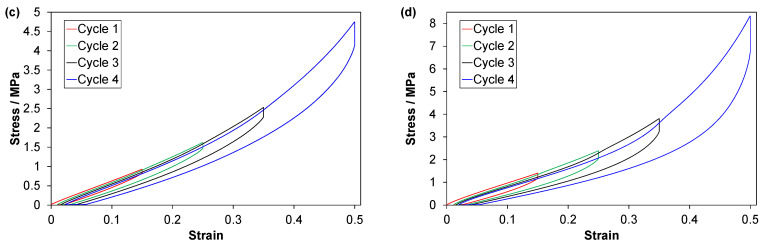
Data from C3 tests for (**a**) TPV, (**b**) LSR, (**c**) EPDM1 and (**d**) EPDM2. Data for one representative test of each material. Note the different ordinate scale for (**d**).

Stress-strain curves from C3 tests are shown in Figure 5. Compared to the C2 tests (Figure 4), there is a vertical segment after the peak stress, due to the stress relaxation stage. There is also a horizontal segment (not seen clearly in Figure 5) at the pre-load force (2 N) after the unloading, which is the strain recovery stage. Compared to C2 tests, the relaxation stage of course affects the unloading curves. The added stages also affect the residual strain prior to reloading, while the peak stress is almost unaffected.

#### 3.2.2. Loading Curves; Modulus, Peak Stress and Mullins Effect

**Figure 6:**
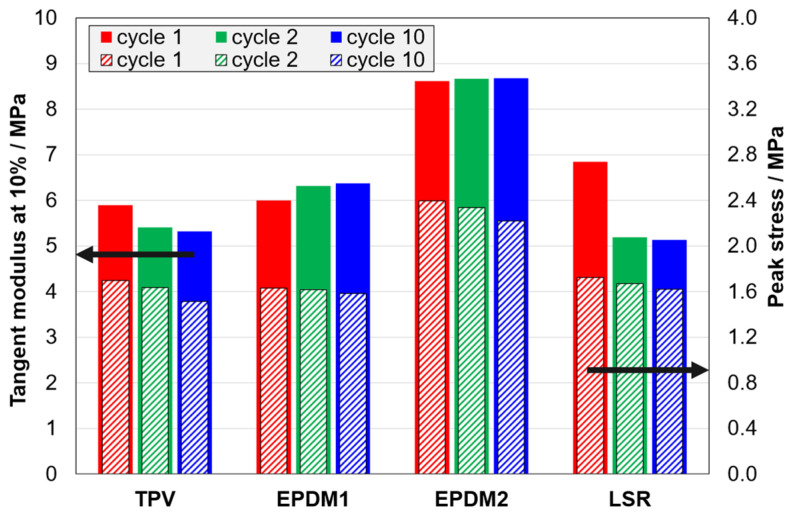
Tangent modulus at 10% strain (solid bars) and peak stress (dashed bars) for C1 tests.

For the loading curves, the peak stress decreases slightly with increasing cycle number (Figure 6), as for most polymeric materials. The shape of the loading curve also changes with cycle number, as reflected in the tangent modulus at 10% strain in Figure 6. Note that there are some patterns which distinguish between the materials: The tangent modulus of the TPV decreases with increasing cycle number, but the opposite is observed for EPDM1. The LSR has a large drop from cycle 1 to cycle 2. The secant moduli at 10% (not shown) show a similar pattern, but with a larger drop from the first to the second cycle for the TPV, and a smaller corresponding drop for the LSR. Further analysis of the loading curves is shown in the form of plots of derivatives in Appendix C, Figure A3.

**Figure 7:**
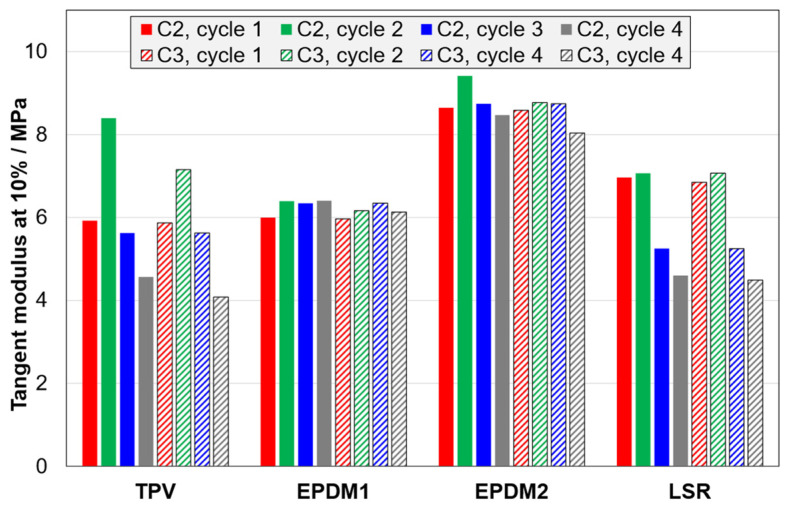
Tangent modulus at 10% strain for C2 tests (solid bars) and C3 tests (dashed bars).

**Figure 8:**
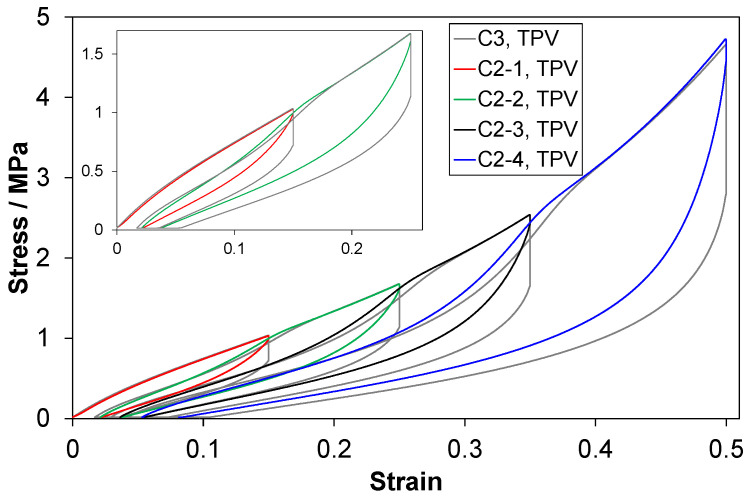
Stress-strain curves for C2 and C3 tests of the TPV. In the inset, only data for the first two cycles are shown.

The next effects that will be considered are the effect of cycle number in C2 and C3 tests (i.e., a combined effect of strain amplitude and cycling), and the effect of including stages with stress relaxation and strain recovery (C3 vs. C2). The 10% tangent modulus only probes one point on the loading curve, but some patterns can be seen: The TPV has a large increase in modulus from the first to the second cycle (15% and 25% strain amplitude, respectively), due to the Mullins effect (Figure 7 and Figure 8).

Furthermore, the TPV and the LSR show a large drop in modulus from the first to the fourth cycle, while the effect is insignificant for the EPDM materials. The stages with stress relaxation and strain recovery have no effect on the peak stress in a given cycle (not shown), but the relaxation has some effect on the loading curve shape. For the tangent modulus at 10%, there is an effect for the TPV (Figure 7 and Figure 8) and a slight effect for EPDM2, but there is no significant effect for the other two materials.

In the literature, the Mullins effect is typically assessed from two consecutive loading curves (*n* and *n* + 1) in a C2 type test, or from the first loading curve and the final “stabilized” loading curve in a C1 type test [28]. However, most studies do not quantify the Mullins effect in C2 type tests. A simple measure of the Mullins effect could be the index *MI*_0_ defined in Table 3. However, when comparing two different materials, *MI*_0_ may also be affected by the different residual strains of the materials. Moreover, it may also be relevant to assess the Mullins effect on a larger portion of the curve segment of loading *n* + 1, below the maximum strain of loading *n*. (Some elastomers have very small *MI*_0_, but still a strong Mullins effect during reloading.) Hence, the two Mullins indices *MI*_1_ and *MI*_2_ are suggested (see Table 3 for definitions). Note that all the three indices are in some way normalized with regard to the strain amplitude of a given cycle.

**Figure 9:**
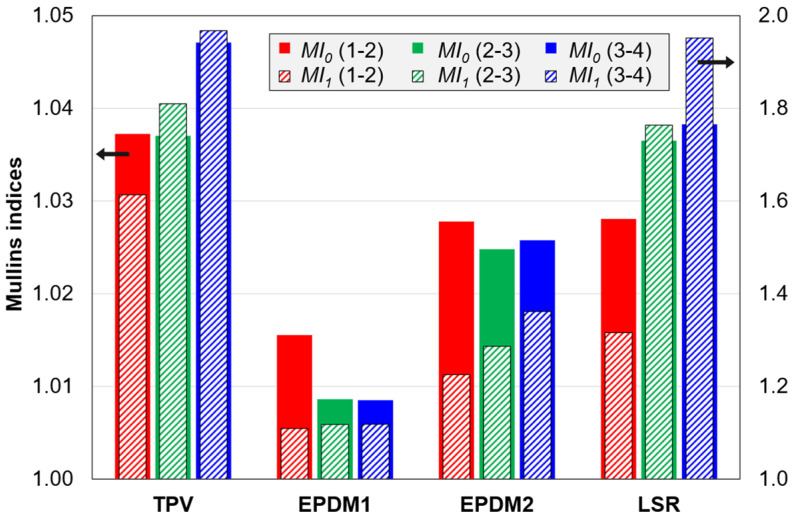
Mullins indices *MI*_0_ and *MI*_1_ (defined in Table 3) calculated from C2 tests. In the legend, the numbers in parenthesis denote the loading curve pair used in the calculation.

Values for *MI*_0_ and *MI*_1_ are shown in Figure 9. The main trend is that the TPV has the largest indices, followed by the LSR, EPDM2 and EPDM1. However, for the loading curve pairs 2–3 and 3–4, the difference between the TPV and the LSR is small. The two indices show slightly different trends. For the TPV and the LSR, *MI*_1_ is more sensitive to cycle number/strain amplitude than *MI*_0_, while the opposite is observed for EPDM1. For the EPDM materials, the two indices even show opposite trends from curve pair 1–2 to 2–3.

Values for *MI*_1_ and *MI*_2_ are compared in Appendix C, Figure A4. These two indices are highly correlated, but *MI*_2_ cannot be used for cases which exhibit very weak Mullins effects, as explained in the caption of Appendix C, Figure A4. An alternative to *MI*_2_ is presented in Appendix D.

#### 3.2.3. Compression Set and Hysteresis Loss

**Figure 10:**
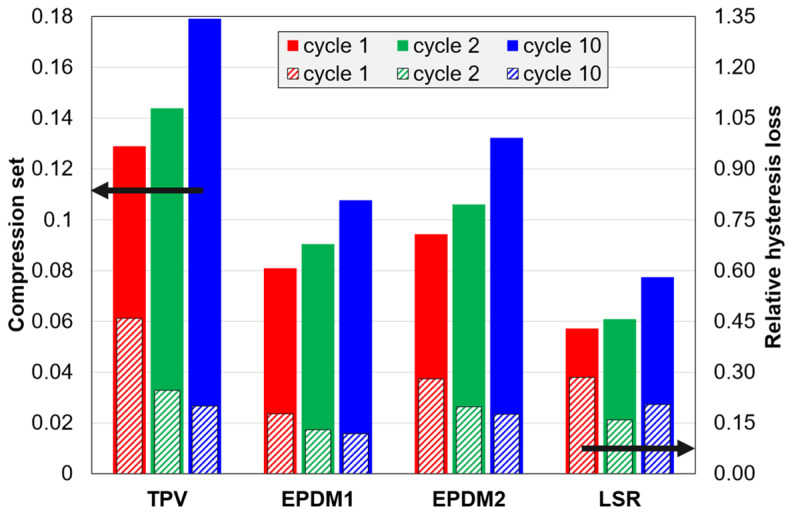
Compression set and relative hysteresis loss for C1 tests.

Values for the compression set immediately after unloading (CS defined in Table 3), for C1 tests, are shown in Figure 10. For all materials, the compression set increases with increasing cycle number. The TPV has the largest values, and the LSR has the lowest values. Figure 10 also shows relative hysteresis losses. Three of the materials show the expected trend; the hysteresis loss decreases with increasing cycle number. However, the LSR has a larger loss in cycle 10 than in cycle 2. This is due to the anomalous loading curves of this material; the loading curves for different cycles overlap in a certain strain range, while the unloading curves shift horizontally to larger strains with increasing cycle number, especially at intermediate and high strains; see Figure 3b.

**Figure 11:**
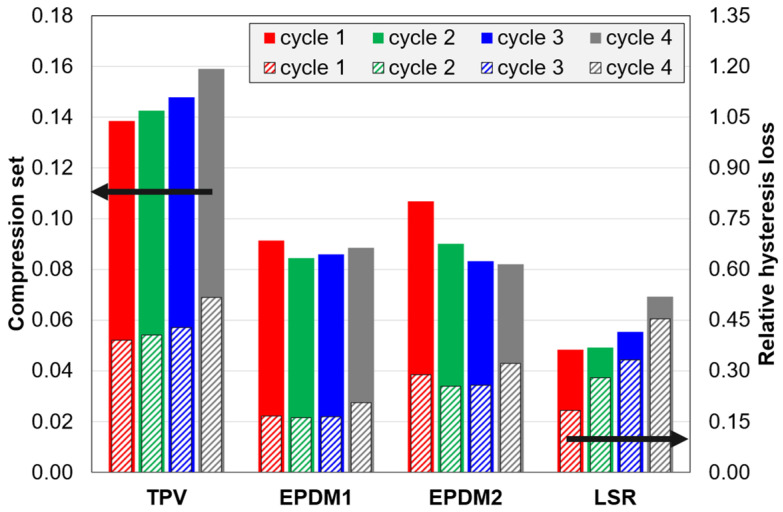
Compression set and relative hysteresis loss for C2 tests.

Regarding compression set vs. cycle number/strain amplitude in C2 tests (Figure 11), there is an increasing trend for the TPV and the LSR, while the compression set is almost constant for EPDM1, and decreases in the three first cycles for EPDM2. For the hysteresis loss in Figure 11, there are similar trends for the TPV and LSR vs. the EPDM materials. Note that the absolute hysteresis loss (Appendix C, Figure A5a) increases with increasing strain amplitude for all materials in C2 and C3 tests. Finally, it can be observed that, among these materials, the LSR has the lowest ratio of compression set to relative hysteresis loss.

**Figure 12:**
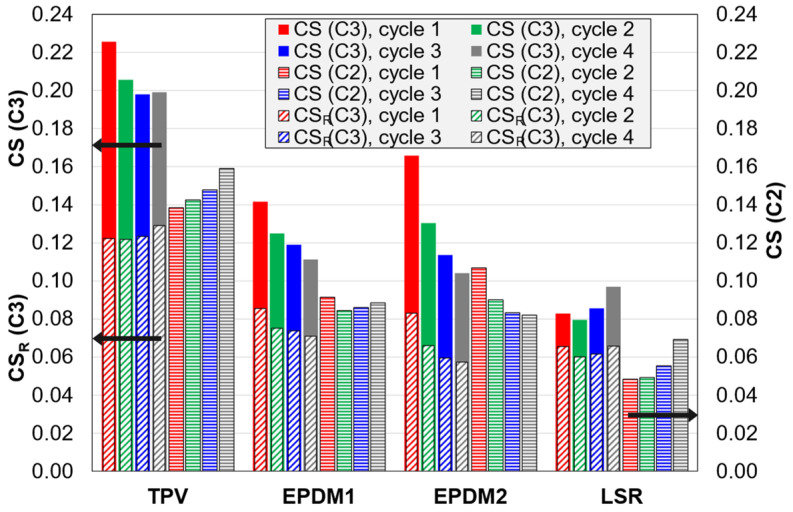
Compression set data for C2 and C3 tests. *CS* is the compression set instantly after unloading, and *CS_R_* is the compression set after the strain recovery stage in C3 tests.

The period with constant strain (stress relaxation) before unloading gives an increase in the compression set for all materials (*CS* for C3 vs. C2 tests in Figure 12). The “graphical” explanation is that for C3 tests the unloading starts from a “lower position”, and the different shape of the unloading curve in C3 vs. C2 only partly compensates for this. The trends for compression set vs. strain amplitude (cycle number) are also affected by the stress relaxation. For the TPV, the trend changes from increasing (for C2) to decreasing (for C3). For EPDM1, it changes from almost constant to a decreasing trend. For EPDM2, it is decreasing in both cases. For the LSR, it is increasing in both cases. Note that only the LSR shows an increasing trend for the C3 tests.

The period with strain recovery after unloading naturally reduces the compression set after recovery (*CS_R_* vs. *CS* for C3 tests in Figure 12). For the TPV and the EPDMs, the strain recovery reduces the compression set values (*CS_R_* of C3) below the values for C2 tests (*CS* of C2). However, this is not the case for the LSR.

Finally the ratio of the *CS* values of the LSR and EPDM1 for C3 tests can be compared. For *CS* (instantly after unloading), this ratio increases monotonously vs. cycle number (strain amplitude) from 0.58 (for cycle 1) to 0.87 (for cycle 4), i.e., the difference between the LSR and EPDM1 decreases with increasing strain amplitude. For *CS_R_* the trend is the same, and the ratios increase monotonously from 0.77 to 0.93, i.e., the difference between the two materials is smaller after recovery. Finally, if strain recovery curves are fitted with a stretched exponential (Section 3.2.5), and the extrapolated strains in the limit t→∞ are used, the ratios are even higher, increasing monotonously from 0.81 to 0.97. Hence, for the highest strain amplitude in this study (0.5) and long recovery times, the difference between the two materials is very small.

#### 3.2.4. Stress Relaxation and Strain Recovery (C3 Tests)

**Figure 13:**
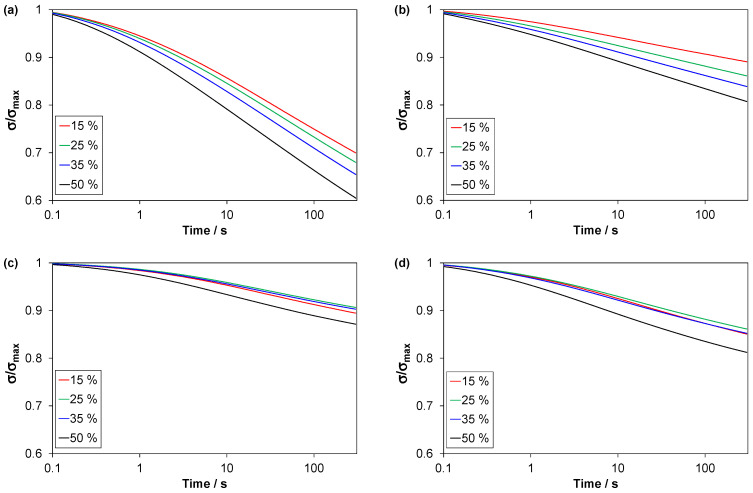
Normalized stress relaxation; stress versus time for the 5 min relaxation stages in C3 tests. Strain amplitudes of the respective cycles are given in the legends. (**a**) TPV, (**b**) LSR, (**c**) EPDM1 and (**d**) EPDM2.

**Figure 14:**
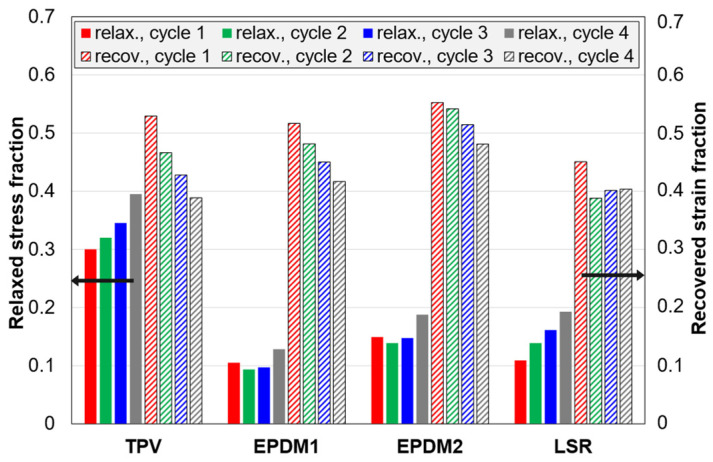
Data from the 5 min relaxation and recovery stages in C3 tests. The relaxed stress fraction is the relaxed stress divided by the stress before the relaxation stage (i.e., end of loading). The recovered strain fraction is the recovered strain divided by the strain before the recovery stage (i.e., end of unloading).

The amount of stress relaxed in the relaxation stage, after loading in C3 tests, increases with increasing strain amplitude (cycle number), i.e., increasing stress at the start of the relaxation stage (stress relaxation curves are shown in Appendix C, Figure A6). In this section, the focus is on the normalized stress relaxation curves (Figure 13) and the corresponding fraction of relaxed stress (Figure 14).

The fraction is highest for the TPV and lowest for EPDM1. For all materials, the fraction is highest for the last cycle. For the EPDMs, the fraction is almost constant for the first three cycles, while it increases steadily for the two other materials. The LSR and EPDM1 have almost equal fractions in the first cycle, but in later cycles the fraction is higher for the LSR. Note that, among these materials, the LSR has the lowest compression set (Figure 12), but its average stress relaxation fraction for all cycles is higher than that of EPDM1, and similar to that of EPDM2.

**Figure 15:**
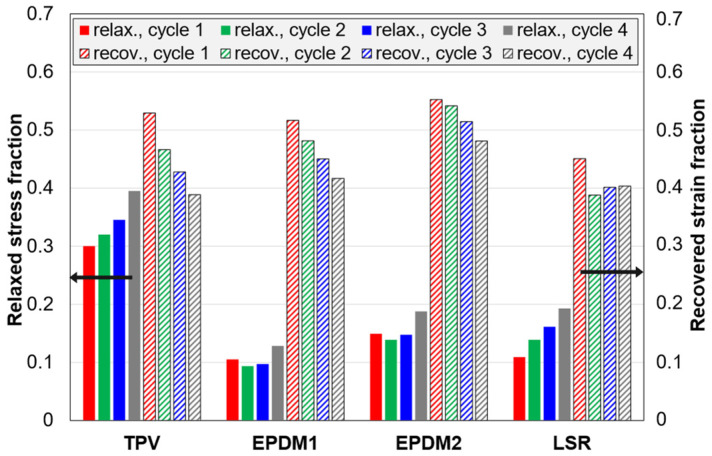
Relaxation rate for normalized stress relaxation and recovery rate for normalized strain recovery, at the end of the respective stages (t = 5 min) in C3 tests. The rates are the derivatives of fitted stretched exponential functions.

The relaxation rates at the end of the relaxation stage (t = 5 min) are shown in Figure 15. As for the relaxed fraction after 5 min (Figure 14), the TPV has the highest value for the rate after 5 min, while the differences between the other materials are small. For all materials, this rate drops from the first to the second cycle. For the TPV and the LSR, the rate increases steadily from cycle 2 to 4, while for the EPDMs, the rate is roughly the same in these cycles.

**Figure 16:**
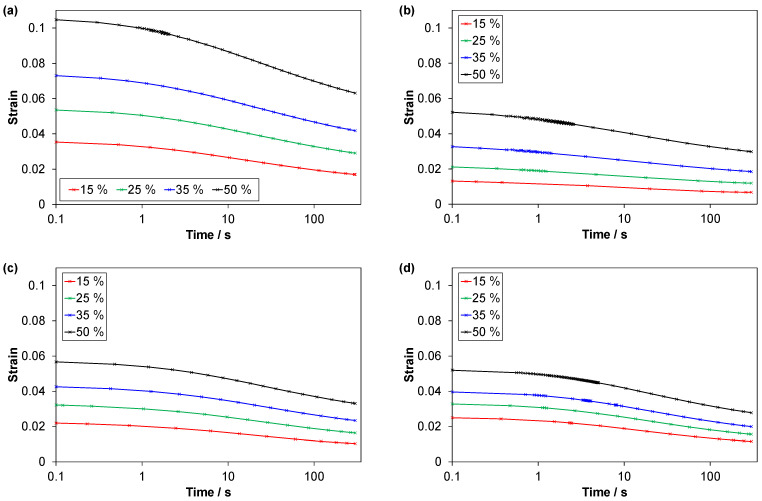
Strain recovery; strain vs. time in the 5 min recovery stages (with pre-force 2 N) in C3 tests. (**a**) TPV, (**b**) LSR, (**c**) EPDM1 and (**d**) EPDM2.

The strain recovery curves, for the 5 min recovery stage with constant low pre-stress after unloading, are shown in Figure 16. There are differences between the materials regarding the strain levels and the effect of strain amplitude.

The recovered strain fractions (defined in Table 3) are shown in Figure 14. The differences between the materials are small. In particular, the TPV and EPDM1 have very similar results. EPDM2 has a slightly higher fraction than EPDM1 for all cycles. For all materials, the fraction is highest for the first cycle. The TPV and the EPDMs show a trend of decreasing fraction with increasing cycle number (strain amplitude), while, for the LSR, the fraction is almost constant for the three last cycles.

The recovery rates of the normalized strains (i.e., normalized by the strain at the start of the recovery), at the end of the recovery stage (t = 5 min), are shown in Figure 15. While the materials have quite similar recovered fractions after 5 min (Figure 14), they have quite different rates. In particular, the LSR has distinctly lower rates than the other materials. EPDM1 has the highest rates.

#### 3.2.5. Fitting Parameters for the Stress Relaxation and Strain Recovery

The stress relaxation, during the 5 min period with constant strain in the C3 tests, was fitted with a stretched exponential function. It was fitted to the absolute stress, as well as the normalized stress (starting at unity at t = 0; the starting point was determined as part of the fitting procedure). The function which was fitted to the stress relaxation is shown in Equation (2). The coefficient of determination (R^2^) was above 0.999. However, the three parameters in this function were correlated in these fits. (The fits with three free parameters are shown in Appendix C, Figure A7.) Hence, in order to simplify the interpretation, the parameter *β* was fixed to the average for all materials and cycles. The two remaining free parameters were then *ϕ* (the relaxed fraction in the limit *t*→∞) and *τ* (the time constant).

(2) $$\sigma \left(t\right)=1-\phi +\phi e^{-{\left(\frac{t}{\tau }\right)}^{\beta }}$$

**Figure 17:**
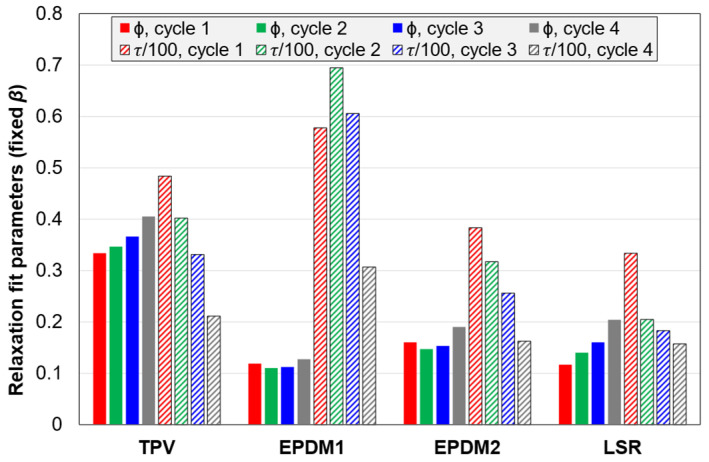
Parameters of Equation (2) fitted to the normalized stress relaxation. The parameter *β* was fixed to the average for the 16 fits with all three parameters free (*β* = 0.422); see Appendix C, Figure A7.

Parameters for the fits with fixed *β* (= 0.422) for normalized stresses are shown in Figure 17. The trends for *ϕ* are close to those of the relaxed stress fractions in Figure 14. Regarding the *τ* values, these are highest for EPDM1 and lowest for the LSR. For three of the materials (EPDM2, LSR and TPV), *τ* decreases with increasing cycle number, while the *τ* of EPDM1 decreases from cycle 2 to 4. The LSR has a more moderate change in *τ* values from cycle 2 to 4 than the other materials. If *β* is fixed to the average for EPDM1 (0.532), EPDM1 also shows the trend of *τ* decreasing with cycle number. Hence, for the normalized stress relaxation, there seems to be a trend that the time constant *τ* decreases with increasing strain amplitude (and stress level). For the absolute stress, *τ* follows somewhat different trends from cycle to cycle, but one trend for both normalized and absolute stress is that the LSR shows the least variation in *τ* from cycle 2 to 4.

**Figure 18:**
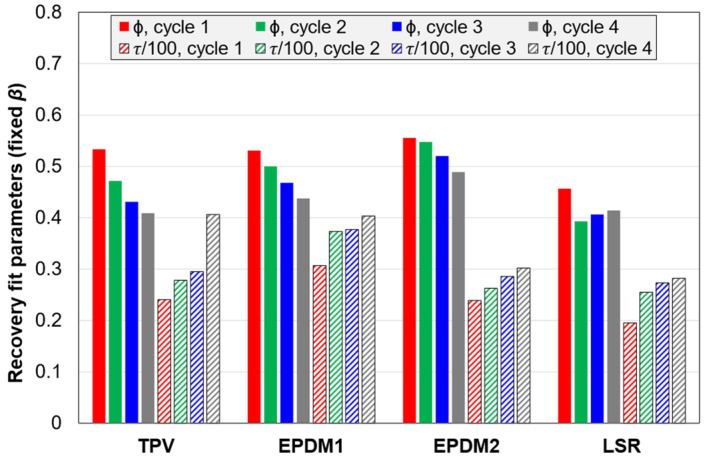
Parameters of Equation (2) fitted to normalized strain recovery. The parameter *β* was fixed to the average for the 16 fits with all three parameters free (*β* = 0.538); see Appendix C, Figure A8.

Similar fits, with a stretched exponential function with fixed *β,* were performed for the normalized strain recovery of C3 tests (Figure 18). Fits with three free parameters are shown in Appendix C, Figure A8. As expected, the parameter *ϕ* is closely related to the fraction of strain recovered after 5 min. Regarding the *τ* values, there is less variation between materials and from cycle to cycle than for the normalized stress relaxation.

#### 3.2.6. Poisson’s Ratio

**Figure 19:**
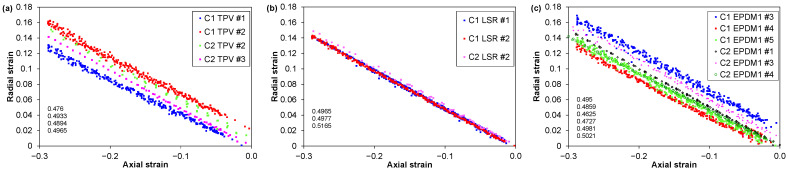
Optically measured radial strain versus axial strain for (**a**) TPV, (**b**) LSR and (**c**) EPDM1. The absolute values of the slopes of linear fits are indicated. Loading and unloading data for 3 to 6 repeats of each material, for up to 10 cycles (C1 test) or 3 cycles (C2 test).

The optically measured radial strains in C1 and C2 tests are shown in Figure 19. The four materials have similar Poisson’s ratios close to 0.5; see Table 5. There is no clear trend vs. cycle number; subsequent cycles appear to coincide with previous cycles. A small vertical shift is seen between the loading and unloading curves (a somewhat larger radial strain was measured during loading than during unloading). Student t-tests indicate 73% statistical similarity between the Poisson’s ratios of the TPV and EPDM1, but only about 10% similarity between the LSR and the TPV or EPDM1.

### 3.3. Tensile Tests to Large Strains

**Figure 20:**
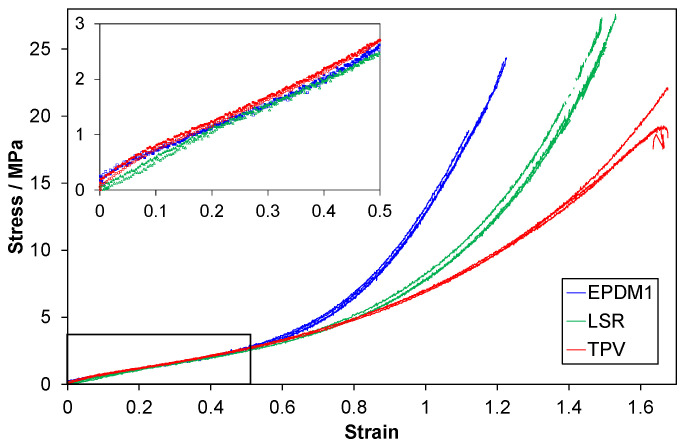
Cauchy stress versus Hencky strain for tensile tests. Three repeats per material are shown. The Cauchy stress calculation is based on a constant Poisson’s ratio (from DIC strain measurements on the front face of the specimen). The strain in the diagram is the axial strain measured by DIC.

The results of tensile tests to failure are shown in Figure 20. Cauchy stress versus Hencky strain (true stress vs. true strain) in Figure 20 shows that the materials have quite similar stress responses up to a strain of 0.5. Beyond this strain, the EPDM1 stress increases relative to that of the LSR and the TPV. At a strain of 0.8, a similar “stress upturn” occurs for the LSR, relative to the TPV.

## 4. Discussion

### 4.1. Overview

The three materials with similar hardness and peak stress (for a certain strain amplitude), but with different chemical compositions, polymer networks and fillers, showed distinctly different responses in cyclic compression tests, as shown in detail by the various parameters derived from the stress-strain curves. The main trends for the TPV vs. EPDM1 were as expected (the TPV having higher compression set, higher hysteresis loss, etc.). However, details have been added to the picture by some observations related to stress relaxation and strain recovery in C3 tests, and how certain properties vary with strain amplitude in C2 and C3 tests. Regarding the LSR, the trends vs. the other materials were more complex. The LSR had the lowest compression set, but for other properties EPDM1 was better (lower hysteresis loss, smaller Mullins effect, etc.). Furthermore, for the compression set after unloading and after strain recovery in C3 tests, the difference between the LSR and EPDM1 decreased with increasing strain amplitude, and when extrapolating to infinite recovery times, the difference was only a few percent. The differences between EPDM1 and EPDM2 (with slightly different hardness values), were likely related to differences in crosslink density and/or carbon black fraction.

### 4.2. Mullins Effect

The mechanism(s) for the Mullins effect are not well understood [11], and the details are material dependent. For a test like C2, it is difficult to distinguish clearly between a Mullins effect (non-permanent damage) and effects of time dependency and permanent damage. One way to interpret the experimental data would be to fit a viscoelastic model combined with a Mullins model [31]. Furthermore, the type of test must be specified when comparing Mullins data. A test like C2 is typically used. However, note that the Mullins effect observed when comparing, e.g., the 3rd and 4th loading in the C2 test will be different from the effect in a test in which the same strains were applied in the 1st and 2nd cycle. Hence, the loading history must be considered. For the C2 test, when comparing a Mullins index for cycle 1 to 2 with an index for cycle 2 to 3, it is a combined effect of strain amplitude and cycling.

Regarding the three different Mullins indices in this study, *MI*_0_ is perhaps the one with the most direct coupling with the residual strain (although the correlation is not high; see next subsection). As an example, the EPDMs having the highest *MI*_0_ value for the 1–2 pair may be an effect of the residual strain. For later pairs, the difference between the starting points of loading curve *n* + 1 and *n* is smaller, and the contribution of this difference to the *MI*_0_ value would be smaller. The index *MI*_2_ had the drawback that it could not be calculated for very weak Mullins effects. Hence, *MI*_1_ is perhaps the best of these indices.

The TPV and the LSR showed a clear Mullins effect—strongest for the former (C2 tests, Figure 4 and Figure 9). The EPDMs showed a weak Mullins effect, especially EPDM1. There are also effects of cycle number/strain amplitude (in the C2 test).

There are only a few studies addressing the Mullins effect in TPVs [22,23,32], and some that model the micromechanics in TPVs [33,34]. Wang and co-workers [22,23,32] observed a softening upon reloading which increased with increasing thermoplastic fraction and increasing strain amplitude. The authors referred to this as a Mullins effect, but it is not a pure (recoverable) Mullins effect. The softening during reloading was explained by this mechanism: The first loading leads to a permanent deformation (yielding) of a small fraction of the thermoplastic matrix, and in the second loading the deformation mainly occurs in the soft rubber phase. As stated in the first paragraph, it is difficult to clearly distinguish the Mullins effect from other effects, especially for elastomers such as TPVs which have a non-crosslinked matrix.

The Mullins effect for the LSR was clear in the C2 tests (Figure 4 and Figure 9). The LSR used in this study showed a stronger Mullins effect than reported in ref. [26], and less than in ref. [27]. This could be because the LSR in ref. [26] was softer (tangent modulus about 2.5 MPa compared to the LSR investigated in this study with 7 MPa) and ref. [27] tested in tension. The Mullins effect in the LSR investigated in this study is likely to be related to a weak interaction between the filler and polymer network. Hanson et al. [12] proposed that the Mullins effect in silica-filled PDMS was due to chain entanglements being removed by one chain sliding under another chain at its attachment point to a silica particle. The silica fraction in the LSR investigated in this study is likely lower than the carbon black fraction in the EPDMs, but silica particles are more difficult to disperse than carbon black particles [5,6]. Note that the Mullins effect in a rubber would increase with reduced crosslink concentration [35], but this would also increase the compression set. Hence, a low crosslink concentration is not a likely explanation for the Mullins effect in the LSR investigated in this study.

**Figure A3:**
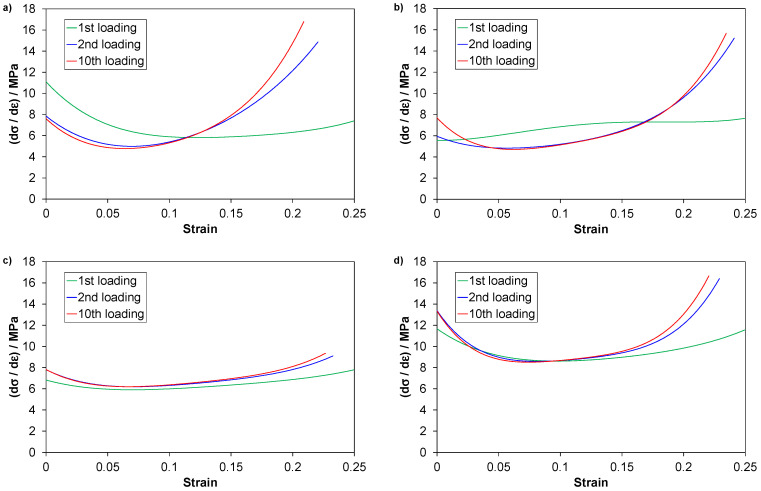
Derivative of stress with regard to strain for C1 loading curves. The zero strain for a given curve in the figure is obtained by extrapolating the corresponding stress-strain curve to zero stress. (**a**) TPV, (**b**) LSR, (**c**) EPDM1 and (**d**) EPDM2.

**Figure A4:**
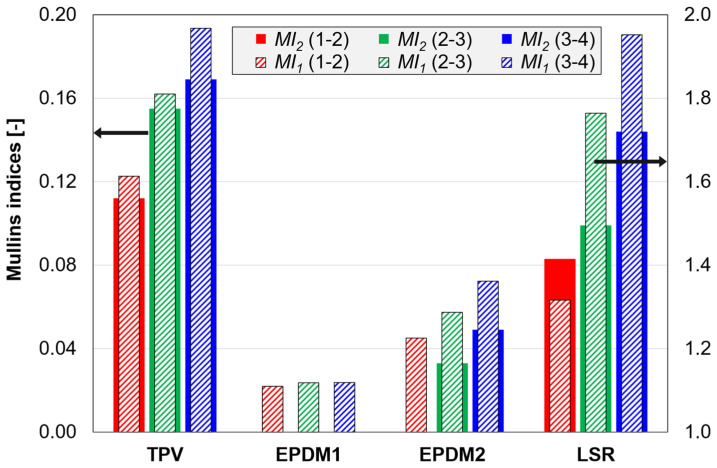
Mullins indices *MI*_1_ and *MI*_2_ (defined in Table 3) calculated from C2 tests. In the legend, the numbers in parenthesis denote the loading curve pair used in the calculation. Regarding *MI*_2_ for EPDM1: This index is zero because the “loop area” between the shifted curves is zero for the first loading curve pair (1–2), or essentially zero for the other pairs. For the (1–2) pair the two shifted curves separate from the start (second curve above the first). For the other loading pairs, the two curves overlap up to a certain strain before separating (second curve above the first). The end of the overlap is at a strain of 0.19 for (2–3) and 0.27 for (3–4). (These strains start at zero at the start of the respective loading curves.) Furthermore, the zero value of this index for the first loading curve pair (1–2) of EPDM2 is also due to a zero-overlap area, but with some overlap. In this case, the end of the overlap is at a strain of 0.07.

EPDM1 and EPDM2 showed a weak Mullins effect (smallest for EPDM1) in the C2 tests (Figure 4 and Figure 9). The difference increases with increasing strain amplitude (*MI*_1_ in Figure 9). As the difference appears at moderate strains, it is perhaps related to the different carbon black fractions in EPDM2 and EPDM1, as filler-matrix and filler-filler damages mainly appear above a certain threshold strain [16]. In addition, the effect of strain amplitude on the peak stress shows a larger relative increase in stress between the third and fourth cycle for the EPDM2 than for the EPDM1 (Figure 4). This more pronounced increase for EPDM2 could, however, be due to both network differences (such as higher crosslink density) and different carbon black fractions. Plagge and Klüppel [36] reported effects of the carbon black fraction on the tensile cyclic loading up to 200% strain; the data showed that the upturn in stress during loading is more well-defined and occurs at a lower strain, as the filler fraction is increased. A characteristic of the EPDMs vs. the other materials is that at low strains the derivative of the second loading curve is larger than that of the first loading curve (see Appendix C, Figure A3) and this is perhaps related to reorganization of filler particles.

### 4.3. Compression Set (CS and CS_R_)

Among the four materials, the LSR had the lowest *CS* (i.e., immediately after unloading) in all tests (Figure 10, Figure 11 and Figure 12). This is probably due to a regular network with high crosslink density and few dangling chains, which is typical for an addition-cured LSR [24]. On the other hand, the literature indicates that a peroxide-cured rubber has a less regular network [24], which could explain why EPDM1 had somewhat higher *CS* than the LSR. As expected, the TPV had the highest *CS* in all tests, due to the thermoplastic matrix.

For all four materials, the *CS* increased with cycling in C1 tests (Figure 10). Although the difference between cycle 1 and 10 was largest for the TPV, likely related to its thermoplastic matrix, this difference was also quite large for the three rubbers. The effect of cycling was slightly larger for EPDM2 than for EPDM1, which could be due to the higher carbon black fraction in EPDM2.

The effect of strain amplitude (and cycling) in C2 tests (Figure 11) differed among the materials. For the TPV and the LSR, the *CS* increased with increasing strain amplitude. For the LSR, this is likely due to relatively weak filler-matrix interactions. For the TPV, the increase could be related to the thermoplastic matrix. The trends were different for the EPDMs; EPDM1 showed almost no effect of strain amplitude on the *CS*, while EPDM2 showed a reduction in *CS* with increasing strain amplitude (although the residual strain after unloading increased with strain amplitude also for EPDM2). Hence, among these materials, the EPDMs’ *CS* was less sensitive to the strain level (up to 50%).

For all four materials, the *CS* increased when adding a stress relaxation period (C3 vs. C2 tests, Figure 12), i.e., the relaxation reduced the elastomers’ ability for strain recovery during unloading. As expected, the difference in *CS* between C2 and C3 tests was largest for the TPV. For the TPV it was also observed that the effect of strain amplitude on the *CS* changed from positive for the C2 tests to negative for the C3 test, even though the relaxed stress fraction increased with increasing strain amplitude. Hence, it seems that the effectiveness of the stress relaxation period, in increasing the *CS*, decreased with strain level.

Regarding the compression set values after the strain recovery stage in C3 tests (*CS_R_*, Figure 12), the difference between the TPV and the LSR is smaller than for the *CS* values from C2 tests (Figure 12). Hence, the equalizing effect of the recovery stage seems to dominate over the opposite effect of the relaxation stage. Moreover, when comparing EPDM1 and the LSR, the difference is smaller for *CS_R_* than for *CS* from C2 tests, and for the highest strain amplitude, the two materials have almost the same *CS_R_* values. However, when comparing the TPV and EPDM1, the difference is about the same for *CS_R_* as for *CS* from C2 tests. Hence, again, the LSR has a deviating behaviour.

### 4.4. Relative Hysteresis Loss (HL)

The TPV had the highest *HL*, which probably is related to its thermoplastic matrix. The LSR had a relatively low *HL* value, and EPDM1 had the lowest values in this study.

**Figure A5:**
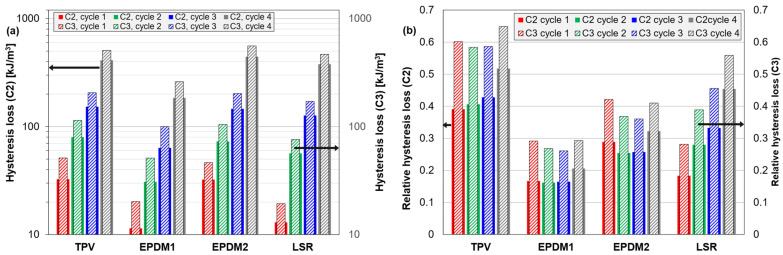
(**a**) Absolute hysteresis loss and (**b**) relative hysteresis loss for C2 and C3 tests.

**Figure A6:**
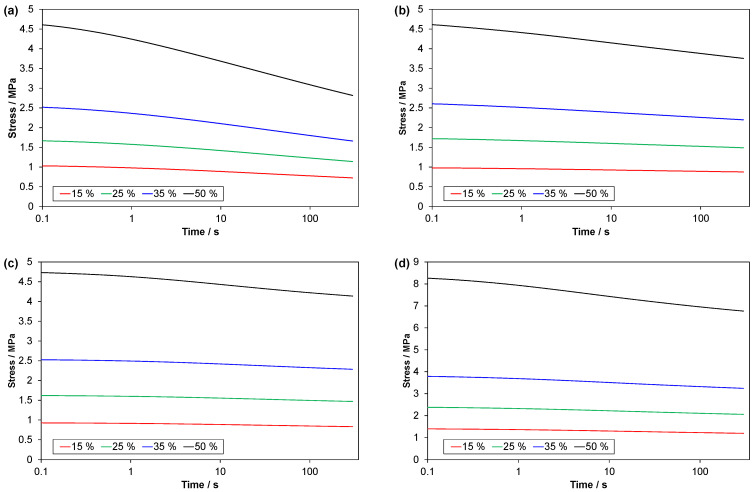
Stress vs. time in the 5 min stress relaxation stage of C3 tests. Time is set to t = 0 s at the beginning of the stage. (**a**) TPV, (**b**) LSR, (**c**) EPDM1 and (**d**) EPDM2.

**Figure A7:**
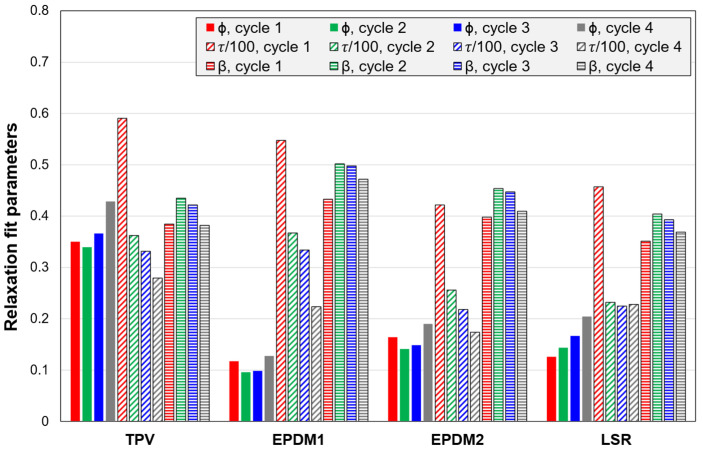
Parameters of stretched exponential function (Equation (2)) fitted to the normalized stress relaxation (C3 tests).

**Figure A8:**
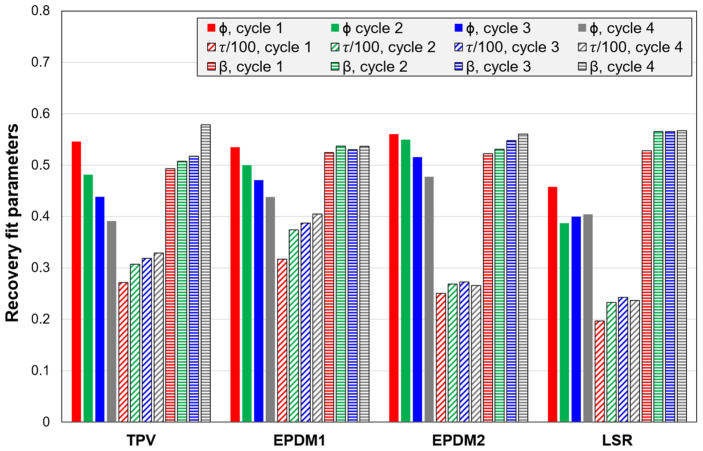
Parameters of stretched exponential function (Equation (2)) fitted to the normalized strain recovery (C3 tests).

For the LSR, the effect of cycling (C1 tests, cycle 2 to cycle 10, Figure 10) deviated from the trend for the other materials by showing an increase in *HL* from the second cycle onwards. (The absolute hysteresis loss showed the same increasing trend; see Appendix C, Figure A5). An increase in *HL* was also observed in ref. [25], but unlike for the LSR investigated in this study, their observation was coupled with a subtle increase in maximum stress. The increase in *HL* for the LSR investigated in this study results from the fact that the loading curve is less affected by cycling than the unloading curve. It was also observed that the hysteresis loop was displaced more at high strains than at low strains. This could be due to a partial recovery of the interaction between filler and polymer network (as well as between filler particles) upon unloading, similar to that suggested in ref. [16].

In contrast to the EPDMs, the relative hysteresis loss of the LSR and the TPV increased with increasing strain amplitude (C2 tests, Figure 11). This increase is mainly caused by the strong Mullins effect of these two elastomers. A similar increase for an LSR was reported in the supporting material of ref. [26].

EPDM1 had lower *HL* than EPDM2 in C1 and C2 tests (Figure 10 and Figure 11). This difference is attributed to a lower carbon black fraction in EPDM1, and perhaps also a higher crosslink density in EPDM2. The EPDMs showed similar trends as C1 tests and C2 tests.

### 4.5. Stress Relaxation and Strain Recovery

The TPV showed the highest relaxed stress fraction (Figure 13a and Figure 14 ) and the highest relaxation rate for the normalized stress at the end of the relaxation stage (Figure 15). These observations can be explained by disentangling and slippage of chains in the thermoplastic matrix [37].

The effect of strain amplitude on the relaxed stress fraction of the TPV is a result of the balance between the responses of the thermoplastic matrix and the rubber particles. At low strains, the stiffer thermoplastic matrix functions as scaffolding, while at larger strains, portions of the matrix are softened by plastic deformation and the overall behaviour becomes increasingly dependent on the contribution from the rubber phase.

For the EPDMs, the relaxed stress fraction did not change much with increasing strain amplitude in the three first cycles, i.e., up to 35% strain (Figure 14), while it increased by each cycle for the LSR and the TPV. The behaviour of the EPDMs may be explained by a strong interaction between carbon black particles and the polymer matrix, and damage only occurring above a certain strain.

The recovered strain fraction (Figure 14) was quite similar for the four materials, with a general trend that the fraction decreased with increasing strain amplitude. For the LSR, however, the fraction was almost the same for cycle 2 to 4. This behaviour of the LSR, compared to the EPDMs, is likely due to the LSR having a more regular network. The LSR had the lowest recovery rate of normalized strain at the end of the recovery stage (Figure 15). This was probably because the LSR was nearer its final residual strain in the limit t→∞, and its viscoelastic strain recovery during unloading was faster than for the other materials, likely due to a more regular network and relatively weak filler-matrix interaction.

### 4.6. Correlation between Parameters from the Cyclic Compression Tests

#### 4.6.1. Compression set (*CS*) vs. Relative Hysteresis Loss (*HL*)

Figure 11 (for C2 tests) shows that there is some correlation between *CS* and *HL* for this dataset of four materials × four cycles. Graphically, a larger residual strain would directly increase the hysteresis loss. Note that the cycle trends for the TPV and the LSR contribute to high correlation, while the opposite is observed for the EPDMs. Further discussions of the correlation between *CS* and *HL* are given in Appendix E.1.

#### 4.6.2. Mullins Indices vs. Compression Set (*CS*) and Relative Hysteresis Loss (*HL*)

The correlation between either of the Mullins indices and *CS* set is low for C2 data, mainly due to the trend-breaking behaviour of the LSR. On the other hand, the correlation between either of the Mullins indices and *HL* is high for C2 data. Details are given in Appendix E.2.

#### 4.6.3. Compression Set vs. Relaxed Stress Fraction

The stress relaxation stage in the C3 tests clearly leads to higher *CS* values than in the C2 tests (without this stage); see Figure 12. The increased *CS* can be related to the stress relaxation as follows. The direct correlation between the difference (*CS*_C3_–*CS*_C2_) and the relaxed stress fraction is low, but other functions of *CS*_C3_ and *CS*_C2_, with fitting parameters, have rather high correlation with the relaxed stress fraction. Further details are given in Appendix E.3.

### 4.7. Tensile Tests to Large Strains

The three elastomers with similar hardness showed very different stress-strain responses at Hencky tensile strains above about 0.5; see Figure 19. A marked increase in stress occurred at a lower strain for EPDM1 than for the LSR. This “stress upturn” occurring at different strains can suggest that the LSR and EPDM1 have different network structures [25]. However, the different type, concentration and dispersion of reinforcing filler (silica or carbon black) can also have an effect. For an unfilled bimodal LSR network, the strain corresponding to a stress upturn is affected by the fraction and length of the short chain segments in the network [25,38]. An increased concentration of carbon black (in an EPDM) can also reduce the strain at which the stress upturn occurs [36]. These effects can be regarded as related, since the carbon black particles have a bridging effect on the immediately surrounding network, thus acting as additional crosslinks [17].

The aim of these tensile tests was to obtain indications regarding the regularity of the networks, especially those of the LSR and EPDM1. However, as a less regular network and an increased fraction of filler could have similar effects on the tensile loading curve, it is difficult to isolate one effect from the other.

## 5. Conclusions

The primary aim of this study was to compare three elastomer types (TPV, LSR and EPDM) regarding their response in cyclic compressive loading. One rationale behind the material selection is that TPV and LSR allow for two-component injection moulding, and, hence, may replace a common rubber such as EPDM in certain applications. Our aspiration is that this study will add insight into the similarities and differences between these elastomer types.

The performance of the TPV was in most cases governed by its thermoplastic matrix. The TPV had the largest Mullins effect, compression set and hysteresis loss, and also the highest stress relaxation. However, in some cases, the difference between the TPV and the other elastomers was not large. Furthermore, for some properties, the research on TPVs will lead to even better TPVs in the future.

The LSR had the lowest compression set in this group. This was probably due to the LSR having a rather regular (uniform) network structure. However, the strong filler-filler interaction in the LSR, in combination with a weak filler-matrix interaction, may explain its larger hysteresis loss and Mullins effect compared to the EPDM materials. In cyclic tests with constant strain amplitude, the relative hysteresis loss of the LSR increased from cycle 2 to 10, while the opposite was observed for the other materials. This hysteresis loss trend for the LSR was due to its loading and unloading curves being non-uniformly shifted to larger strains with increasing cycle number; the shift was largest for the unloading curves.

Hence, when comparing the LSR with the other materials, the LSR in many cases showed deviating trends. These deviations are attributed to the LSR having a somewhat different polymer network architecture and a different filler (silica).

The EPDM materials had a somewhat higher compression set than the LSR, which is likely due to a less regular network. Moreover, the interaction between EPDM and carbon black particles is strong and this is the likely explanation for the low hysteresis loss and the weak Mullins effect.

The EPDM materials differed from the other two materials regarding the effect of strain amplitude on compression set, relative hysteresis loss and relaxed stress fraction. For the EPDM materials there was almost no effect of strain amplitude for amplitudes up to 35%, while both the TPV and the LSR showed an increase in these entities already from 15% to 25%. The difference between the compression set values of EPDM1 and the LSR decreased with increasing strain amplitude and increasing strain recovery time.

Some methods for quantifying the Mullins effect are suggested in this paper, aiming at comparing the strength and character of the Mullins effect for different materials, strain amplitudes and cycles. The calculated Mullins indices agreed with visual assessments of consecutive loading curves. For the dataset comprising tests with stepwise increasing strain amplitude for the four materials, the correlation between any of the Mullins indices and the relative hysteresis loss was high, and the correlation between a Mullins index and the compression set was also high, if the LSR dataset was excluded. Further studies are needed on the relationships between Mullins index, compression set and relative hysteresis loss, for different cyclic test programs and material groups.

## Figures and Tables

**Table 1:** The four elastomer materials in this study. All were black.

Material ID	Material Type ^1^	Grade (Manufacturer)	Hardness ^2^ (Shore A)
TPV	TPV − (xSEBS + PP)	TV6VAZ (Kraiburg TPE, Waldkraiburg, Germany)	62.5
LSR	Liquid silicone rubber, VMQ	Elastosil LR 3070/60 A/B ^3^ (Wacker Chemie, Munich, Germany)	61.8
EPDM1	EPDM	01/U60 EP (LAV.EL. Gomma, Cologne, Italy)	65.2
EPDM2	EPDM	01/U70 EP (LAV.EL. Gomma, Cologne, Italy)	75.1

^1^ For nomenclature for thermoplastic elastomers and rubbers, see standards ISO 18064 and ISO 1629, respectively. TPV = thermoplastic vulcanizate elastomer, xSEBS = crosslinked poly(styrene-b-(ethylene-r-butylene)-b-styrene) copolymer, PP = polypropylene, VMQ = vinyl methyl silicone rubber, (Q class rubber), EPDM = ethylene propylene diene M class rubber. ^2^ Tested according to ISO 48-4:2018, which specifies test durations of 15 s and 3 s, for thermoplastic elastomers and rubbers, respectively. ^3^ This LSR material is a two-part system; one part contains the catalyst and the other the crosslinker. The LSR contained 2% colour paste (FL Black 9005).

**Table 2:** The three cyclical compression tests in this study.

Test ID	Description
C1	Ten cycles with the same strain amplitude (25%). Each cycle has two stages: loading from a small pre-force to the strain amplitude and unloading to the pre-force.
C2	Four cycles with increasing strain amplitude (15%, 25%, 35% and 50%). Loading and unloading stages as for C1 tests.
C3	As C2 tests, but with two additional inserted stages: stress relaxation (at constant strain) for 5 min after the loading stage, and strain recovery (at constant pre-force) for 5 min after the unloading stage.

**Table 3:** Parameters derived for each cycle of the compression tests (test C1–C3).

ID	Parameter	Description
*TM*	Tangent modulus at 10% strain	In this case, the starting point for the 10% strain is the start of the given loading curve, as in ISO 7743. The start point (zero strain for the given loading curve) is obtained by extrapolating the first part of the loading curve to zero stress.
*MI*_0_	Mullins index 0 (from C2 tests)	This index quantifies the Mullins effect as the ratio of the stress in loading n to the stress in loading *n* + 1, at the maximum strain of loading *n*.
*MI*_1_	Mullins index 1 (from C2 tests)	This index quantifies the Mullins effect as the derivative of loading curve *n* + 1 divided by derivative of loading curve *n*, at the strain corresponding to a local maximum of the former derivative, occurring just below the maximum strain of the latter. See Appendix B, Figure A1.
*MI*_2_	Mullins index 2 (from C2 tests)	This index is based on two consecutive loading curves in C2 tests. To reduce the effect of compression set on the index (via the different starting strains for the two loading curves), the curves are shifted horizontally to start at zero strain; see Appendix B, Figure A2. Then, if a Mullins effect is present, the two curves will cross again at a strain somewhat before the end of the first curve. The index is defined as the relative difference between the integrals of the first and second curve, integrated from zero strain to the crossing strain. Here, “relative” means dividing by the integral of the first curve.
*CS*	Compression set after unloading	Residual strain (instantly) after unloading divided by the strain amplitude (before unloading). The residual strain after unloading is obtained by extrapolating the last part of the unloading curve to zero stress.
*CS_R_*	Compression set after recovery (for C3 tests)	Residual strain after the recovery stage, divided by the strain amplitude (before unloading).
*HL*	Relative hysteresis loss	The hysteresis loss is the difference between the integral of the loading curve and the integral of the corresponding unloading curve. The relative hysteresis loss is the hysteresis loss divided by the integral of the loading curve.
*ϕ_σ_*	Relaxed stress fraction (C3 tests)	Difference between the initial and final stress in the 5 min relaxation stage, divided by the initial stress. The initial stress is the same as the peak stress (after loading).
*ϕ_ε_*	Recovered strain fraction (C3 tests)	Difference between the initial and final strain in the 5 min recovery stage, divided by the initial strain. The initial strain is the instant residual strain after unloading. (The initial and final strains are the basis for *CS* and *CS_R_*, respectively.)

**Table 4:** DSC and TGA results. The weight fractions (w_i_) are those defined in ISO 9924-3.

ID	T_g_ (°C)	T_m_ (°C)	H_m_ (J/g)	w_2_ ^a^	w_5_ ^b^	w_7_ ^c^	w_8_ ^d^	T_95%_ (°C)
TPV	−52.9	147.3	17.0	92.9%	1.1%	0.0%	6.0%	280
LSR		−45.3	7.7	- ^e^	0.0%	- ^e^	0.0%	460
EPDM1	−52.9	52.3	2.8	57.0%	35%	1.8%	6.0%	390
EPDM2	−54.1	52.0	1.6	52.4%	41%	0.6%	4.8%	378

^a^ Pyrolyzed polymer (wt% from TGA); ^b^ carbon black (wt% from TGA); ^c^ decomposed minerals (wt% from TGA); ^d^ ash/residue (wt% from TGA); ^e^ refer to main text, Section 3.1.

**Table 5:** Poisson’s ratios determined from C1 and C2 tests (based on the tests shown in Figure 19, axial strains in the range 0–35%).

ID	*ν*	*n*
TPV	0.49 ± 0.01	4
LSR	0.50 ± 0.01	3
EPDM1	0.49 ± 0.02	6

**Figure A1:**
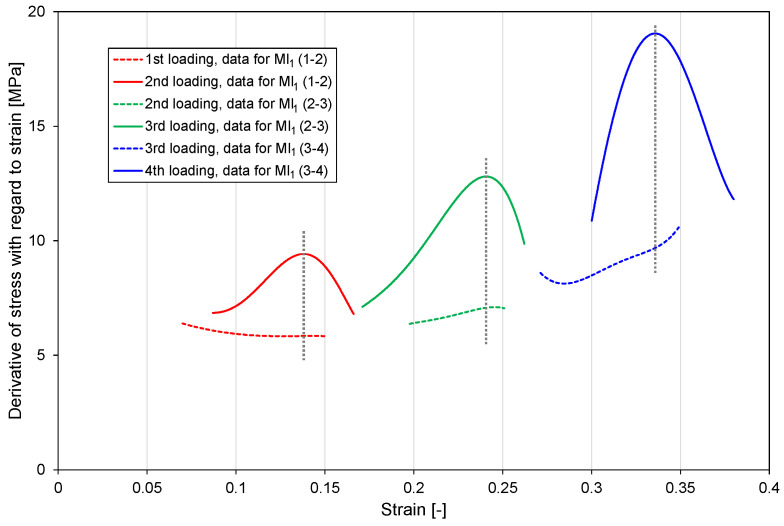
Diagram explaining the calculation of Mullins index 1 (*MI*_1_), based on C2 data for the TPV. This index is calculated as the derivative of loading curve *n* + 1 divided by derivative of loading curve n, at the strain corresponding to a local maximum of the former, which occurs just below the maximum strain of the latter. In the example above, *MI*_1_ for the first and second loading curves (referred to as “*MI*_1_ (1–2)” in the legend and in the article) is the ratio of the solid red curve to the dashed red curve at the strain indicated by the vertical dotted grey line. Then, *MI*_1_ (2–3) is calculated from the green lines, etc. The derivatives are calculated analytically from the polynomials fitted to the individual loading curves (raw data). In this case, only a short segment was fitted, in order to obtain accurate index values.

**Figure A2:**
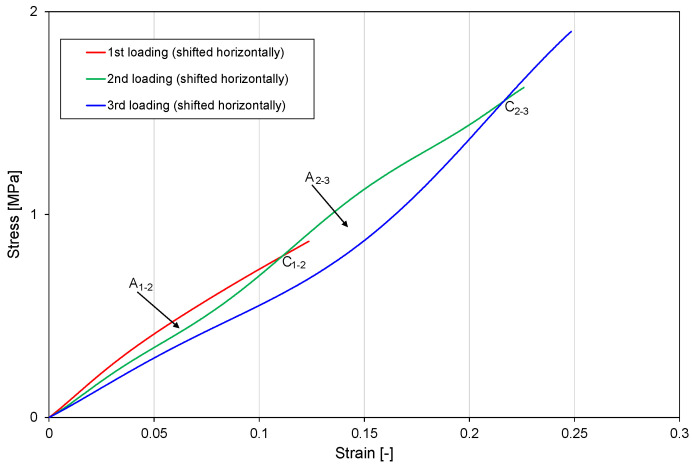
Diagram explaining the calculation of Mullins index 2 (*MI*_2_)-based data for the TPV. This index is based on two consecutive loading curves in C2 tests. First, the curves are shifted horizontally to start at zero strain. Then, if a Mullins effect is present, the two curves will cross again at a strain somewhat before the end of the first curve. The index is defined as the relative difference between the integrals of the first and second curve, integrated from zero strain to the crossing strain. Here, “relative” means dividing by the integral of the first curve. In the example above, *MI*_2_ for the first (red) and second (green) loading curves (referred to as “*MI*_2_ (1–2)” in the legend and in the article) is equal to the area enclosed by the red and green curves (A1–2) divided by the integral of the red curve up to the intersection point (C1–2). Similarly, *MI*_2_ (2–3) is calculated from the green and blue curves. For clarity, the entire loading curves are not shown.

**Figure A9:**
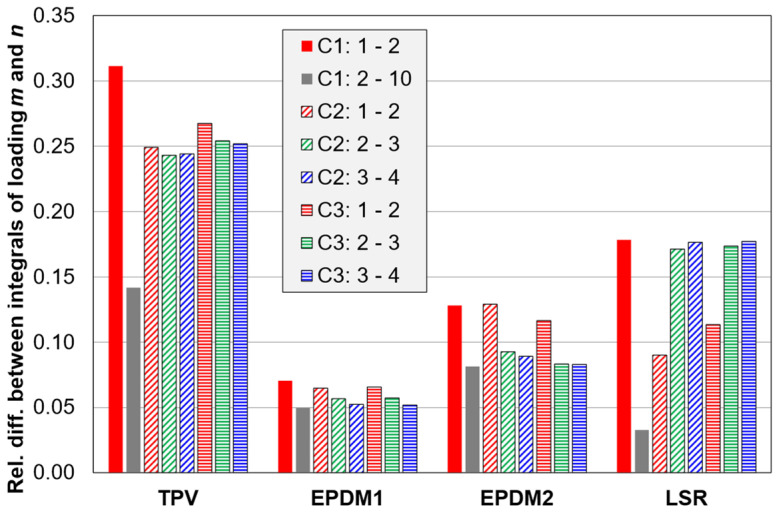
Relative difference between integrals of two loading curves for a certain test. Example: The first entry in the legend is the difference between the integrals of the 1st and 2nd loading of test C1, divided by the integral of the 1st loading. For C2 and C3 tests, with stepwise increasing strain amplitude for each cycle, both loading curves are integrated to the end strain of the first, e.g., 0.15 for “C2: 1–2”.

**Figure A10:**
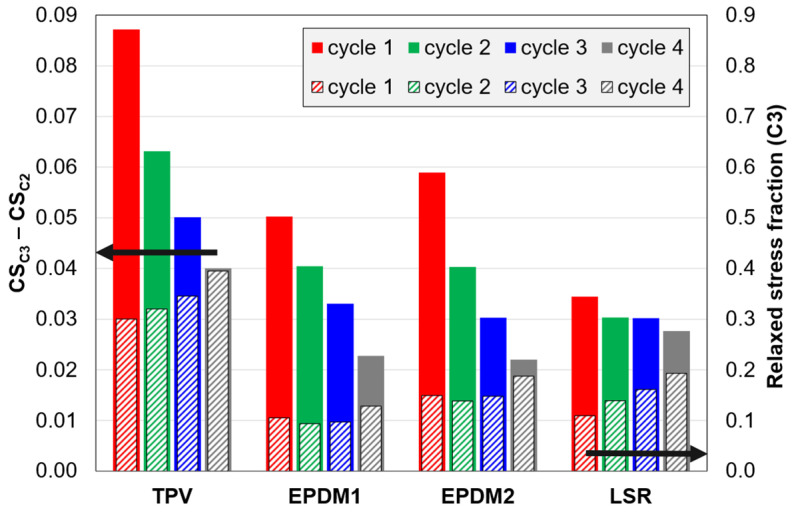
Difference between compression set values (*CS*) in test C3 and C2, compared to the relaxed stress fraction in C3 tests.

**Figure A11:**
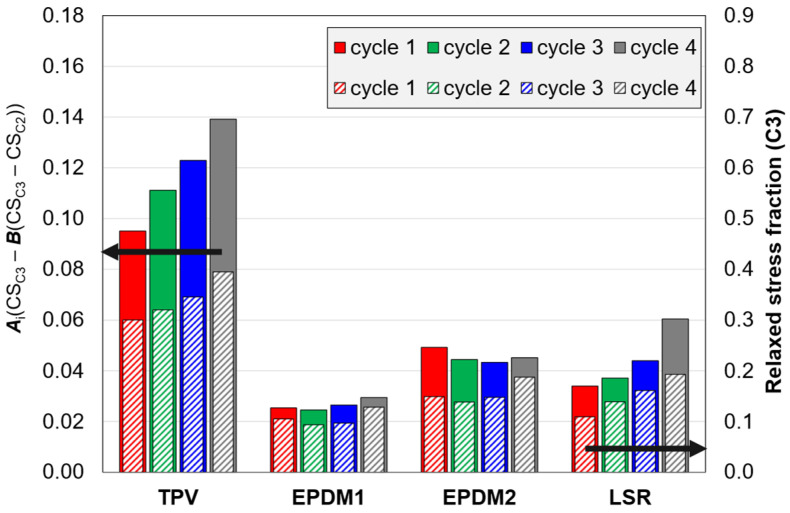
Left axis: a function of compression set values in C2 and C3 tests; see main text for details. In this function, *A_i_* are four fitted material-specific parameters (1.00 (fixed), 0.38, 0.63 and 1.09, respectively, for the materials listed left to right), and *B* is a fitted parameter (1.50).

## Data Availability

Raw test data available in supplementary data.

## Supplementary Materials

The following supporting information can be downloaded at: https://www.mdpi.com/article/10.3390/polym14071316/s1, supplementary S1: data of TPV; supplementary S2: data of LSR; supplementary S3: data of EPDM1; supplementary S4: data of EDPM2.

## Appendix A. Poisson’s Ratio Determined from Axial and Lateral Hencky Strains

A review of nonlinear isotropic elasticity by Mihai and Goriely [39] demonstrated that only the usage of Hencky strain measures produces a constant Poisson’s ratio (*ν*) at large strains.

(A1) $$e_{lat}=-\nu e,$$

where *e* is the longitudinal Hencky strain and *e_lat_* the lateral Hencky strain. Hence, the slope of *e_lat_* versus *e* is −*ν*, and this expression is valid for large strain (i.e., finite strain).

## Appendix B. How Parameters Were Derived from the Compression Stress-Strain Data

### Appendix B.1. Python Script

The data in Section 3.2. were obtained by a Python script which fitted the loading and unloading curves for all cycles individually with polynomial functions of order up to 7 (only the highest necessary order was used). To determine the start of loading curves and the end of unloading curves, via extrapolation to zero stress, only the lower part of the curves were fitted, and the order of the polynomial was reduced in order to avoid oscillations of the polynomials outside the fitting range affecting the extrapolations. The script also corrected all strains for a given test by a small horizontal shift, to correct for the pre-stress, resulting in a non-zero stress at zero strain for the first loading in the raw data. All fits were good (coefficient of determination (R^2^) always above 0.999, and in most cases above 0.9999). It can be noted that some of the unloading curves from the highest strain (50%) were better fitted by a stretched exponential function for the first part of the unloading (about 2/3) in combination with a quadratic function for the last part. However, such fitting functions were not used in this paper.

## Appendix D. Other Parameters for Quantifying the Mullins Effect

An alternative parameter (from C2 tests), which would give values also for cases with weak Mullins effects, could be similar to *MI*_2_, but without the horizontal shifts and just integrating up to the final strain of the first curve. This parameter is partly affected by the curve shape (and Mullins effect) and partly by the starting point (i.e., the residual strain after the preceding unloading), which was attempted to be avoided for the Mullins indices *MI*_1_ and *MI*_2_.

We can normalize this parameter: the relative difference between the integrals of two loading curves. For C1 tests, this can be, e.g., the difference between the integrals of the 1st and 2nd loading, divided by the integral of the 1st loading. For C2 and C3 tests, with stepwise increasing strain amplitude for each cycle, both loading curves are integrated to the end strain of the first curve.

Some results for such relative differences between loading curve integrals are shown in Figure A9. Overall, the TPV has the highest values and EPDM1 the lowest values. For C2 tests, the trend among the materials is the same as for the Mullins indices above, except for the C2 1–2 pair. The trend among the materials for the C1 1–2 pairs also matches that of the Mullins indices. For C1 tests, all materials have a larger relative difference between 1st and 2nd loading than between 2nd and 10th loading. Note that the LSR has the second highest value for the C1 1–2 pair, but the lowest value for the C1 2–10 pair. A comparison between C1 and C2 data also shows some clear differences between the materials. One example is that the 1–2 values for C1 and C2 (integrated to 25% and 15% strain, respectively) are almost the same for the EPDMs, while they are very different for the LSR. Regarding the effect of cycle number for C2 and C3 tests, the LSR shows a deviating trend; the values for the pairs 2–3 and 3–4 are higher than for 1–2.

## Appendix E. Correlation between Parameters from the Cyclic Compression Tests

### Appendix E.1. Compression Set (CS) vs. Relative Hysteresis Loss (HL)

Figure 11 (for C2 tests) shows that there is some correlation between *CS* and *HL* for this dataset of four materials × four cycles. An explanation for this is that, graphically, a larger residual strain would directly increase the hysteresis loss as defined in Table 3. However, the Pearson correlation coefficient, *r*, for the entire dataset in Figure 11 is low (*r* = 0.59), but for the single materials the correlation is higher. If, e.g., the *HL* data for a given material are multiplied by a material parameter, and these four material parameters are optimized to maximize the correlation, this gives *r* = 0.980 (for the 4 × 4 dataset). Hence, this is equal to the average correlation coefficient for the four materials when considered separately. These four fitted material parameters characterize the materials relative to each other. (The values are (1.00, 1.04, 0.69 and 0.17) for TPV, EPDM1, EPDM2 and LSR, respectively. The value for the TPV was set to 1 as normalization.) Alternatively, the *HL* data for a given material can be multiplied by a weight factor equal to the average *CS* for the material, divided by the average *HL* for the material. This gives *r* = 0.956 for the 4 × 4 dataset. Note that the cycle trends for the TPV and the LSR contribute to high correlation, while the opposite is the case for the EPDMs.

### Appendix E.2. Mullins Indices vs. Compression Set (CS) and Relative Hysteresis Loss (HL)

The correlation between either of the Mullins indices and *CS* is low for C2 data. The highest *r* values are obtained when correlating the Mullins index for loading curve pair 1–2 (Figure 9) with *CS* after cycle 2, etc., i.e., a dataset of four materials × three pairs/cycles. The resulting *r* values are 0.42 and 0.41 for *MI*_0_ and *MI*_1_, respectively. However, if the LSR (with large Mullins index and low compression set) is excluded from the dataset, the *r* values increase to 0.834 and 0.939 for *MI*_0_ and *MI*_1_, respectively.

The correlation between either of the Mullins indices and *HL* is high for C2 data. The highest *r* value (0.948) is obtained when correlating *MI*_1_ for loading curve pair 1–2 (Figure 9) with *HL* of cycle 2, etc., i.e., a dataset of four materials × three pairs/cycles.

Hence, apart from the trend-breaking low compression set values of the LSR, the Mullins indices are highly correlated with both *CS* and *HL* for such a 4 × 3 C2 dataset. The former correlation could be expected, as the magnitude of the residual strain is often correlated/linked with the strength of the Mullins effect [11]. The latter correlation indicates that the hysteresis loss is dominated by Mullins damage. Note that all three entities are correlated; Mullins indices, *CS* and *HL*. For comparison with the *r* values cited above, the highest correlation between *CS* and *HL*, for a reduced (four materials × three cycles) C2 dataset, is obtained when correlating *CS* of cycle 2–4 with *HL* of cycle 1–3 (*r* = 0.736). If the LSR data are excluded, the *r* value increases to 0.901. Further work is needed to identify possible unique features of the Mullins effect vs. compression set and hysteresis loss.

Regarding Mullins material models, these account for hysteresis, but many of them do not predict a residual strain, and the unloading curve is equal to the reloading curve. More advanced models do not have these limitations, but many parameters are needed and most of these can only be determined by fitting [11].

Finally, the Mullins “strength” was considered based on the difference between the integrals of the 1st and 2nd loading curves in the C1 test; see Figure A9. (Ref. [28] used the 1st and the stabilized loading curve after a number of cycles. However, the (small) difference between the 2nd curve and the stabilized curve may be affected by other factors than a pure Mullins effect.) The C1 1–2 values in Figure A9 show the same trend among the materials as the *MI*_1_ 1–2 values in Figure 9, but note that the strain amplitudes are different. Other entities in the two figures do not show the same trends, between materials or between cycles.

### Appendix E.3. Compression Set vs. Relaxed Stress Fraction

The stress relaxation stage in the C3 tests clearly leads to higher *CS* values than in the C2 tests (without this stage); see Figure 12. How is the increased *CS* related to the stress relaxation? A first thought would be to relate the *CS* difference (between C3 and C2 tests) to the relaxed stress fraction in the C3 test (see Figure 11), or the absolute relaxed stress. However, the correlation in Figure A10 is low (correlation coefficient *r* = 0.46). In fact, the correlation between the C3 relaxed stress fraction and the C3 *CS* is higher (*r* = 0.78), but still low. Different expressions containing *CS*_C3_ and *CS*_C2_, and various fitted parameters or entities derived from the experimental data, were evaluated with regard to the correlation with the C3 relaxed stress fraction or the absolute relaxed stress. The expression in Equation (A2) gives a good correlation with the relaxed stress fraction.

(A2) $$A_{i}\left[CS_{C3}-B\left(CS_{C3}-CS_{C2}\right)\right]$$

With the parameters *A_i_* set to the average relaxed fraction for all cycles for a given material, i.e., one parameter for each material, and *B* being a fitted parameter, the correlation coefficient is *r* = 0.965. If the parameters *A_i_* are fitted (one for each material), the correlation is improved further (*r* = 0.990), as shown in Figure A11. With fitted *A_i_* parameters, the correlation is also high if *B* is set to zero (*r* = 0.947). However, this best fit gives *A_i_* = 0 for EPDM1, but if *A_i_* is set to 0.1 for EPDM1 (compared to 1 for the TPV), the correlation is still quite high (*r* = 0.943). On the other hand, if all parameters *A_i_* are set to 1 in Equation (A2), the correlation reduces to *r* = 0.84 for the best fit.

## References

[1] Turng L.-S., Osswald T.A., Turng L.-S., Graham P. (2008). Special injection moulding processes. Injection Moulding Handbook.

[2] Persson A.-M.M.R., Hinrichsen E.L., Andreassen E. (2022). On the temperature dependence of the cyclic compressive loading of a thermoplastic vulcanizate elastomer. Polym. Test..

[3] Persson A.-M.M.R., Hinrichsen E.L., Andreassen E. (2020). Adhesion between thermoplastic elastomers and polyamide-12 with different glass fiber fractions in two-component injection molding. Polym. Eng. Sci..

[4] Persson A.-M.M.R., Andreassen E. Ageing effects on two-component injection molded thermoplastic elastomers. Proceedings of the SPE ANTEC 2020.

[5] Dick J.S. (2009). Rubber Technology Compounding and Testing for Performance.

[6] Mark J.E., Erman B., Roland C.M. (2013). The Science and Technology of Rubber.

[7] Drobny J.G. (2014). Handbook of Thermoplastic Elastomers.

[8] Bont M., Barry C., Johnston S. (2021). A review of liquid silicone rubber injection molding: Process variables and process modeling. Polym. Eng. Sci..

[9] Brook M.A., Saier H.-U., Schnabel J., Town K., Maloney M. (2007). Pretreatment of liquid silicone rubbers to remove volatile siloxanes. Ind. Eng. Chem. Res..

[10] Mullins L. (1969). Softening of rubber by deformation. Rubber Chem. Tech..

[11] Diani J., Fayolle B., Gilormini P. (2009). A review on the Mullins effect. Eur. Polym. J..

[12] Hanson D.E., Hawley M., Houlton R., Chitanvis K., Rae P., Orler E.B., Wrobleski D.A. (2005). Stress softening experiments in silica-filled polydimethylsiloxane provide insight into a mechanism for the Mullins effect. Polymer.

[13] Drozdov A.D., Dusunceli N. (2012). Universal mechanical response of polypropylene under cyclic deformation. J. Polym. Eng..

[14] Milani G., Milani F. (2009). Optimal vulcanization of 2D–3D EPM/EPDM thick elements through peroxidic mixtures. J. Math. Chem..

[15] Milani G., Galanti A., Cardelli C., Milani F., Cardelli A. (2015). Combined numerical, finite element and experimental-optimization approach in the production process of medium-voltage, rubber-insulated electric cables vulcanized with steam water. Part 1: DSC and rheometer experimental results. Rubber Chem. Tech..

[16] Candau N., Oguz O., Peuvrel-Disdier E., Bouvard J.-L., Pradille C., Billon N. (2020). Strain and filler ratio transitions from chains network to filler network damage in EPDM during single and cyclic loadings. Polymer.

[17] Litvinov V.M., Orza R.A., Klüppel M., van Duin M., Magusin P.C.M.M. (2011). Rubber–filler interactions and network structure in relation to stress–strain behavior of vulcanized, carbon black filled EPDM. Macromolecules.

[18] Roland C.M. (2016). Reinforcement of elastomers. Reference Module in Materials Science and Materials Engineering.

[19] Liu X., Huang H., Xie Z., Zhang Y., Zhang Y., Sun K., Min L. (2003). EPDM/polyamide TPV compatibilized by chlorinated polyethylene. Polym. Test..

[20] Ren N., Matta M.E., Martinez H., Walton K.L., Munro J.C., Schneiderman D.K., Hillmyer M.A. (2016). Filler-reinforced elastomers based on functional polyolefin prepolymers. Ind. Eng. Chem. Res..

[21] Babu R.R., Singha N.K., Naskar A.K. (2010). Interrelationships of morphology, thermal and mechanical properties in uncrosslinked and dynamically crosslinked PP/EOC and PP/EPDM blends. Express Polym. Let..

[22] Liu Q., Zang K., Zang Z., Wang Z. (2018). Strengthening effect of Mullins effect of high density polyethylene/ethylene-propylene-diene terpolymer thermoplastic vulcanizates under compression mode. J. Thermopl. Compos. Mater..

[23] Wang C.C., Zhang Y.-F., Liu Q.Q., Wang Z.B. (2017). Mullins effect under compression mode and its reversibility of thermoplastic vulcanizate based on ethylene-vinyl acetate copolymer/styrene-butadiene rubber blend. Int. Polym. Proc..

[24] Stricher A.M., Rinaldi R.G., Barrès C., Ganachaud F., Chazeau L. (2015). How I met your elastomers: From network topology to mechanical behaviours of conventional silicone materials. RSC Adv..

[25] Delebecq E., Hermeline N., Flers A., Ganachaud F. (2012). Looking over liquid silicone rubbers: (2) Mechanical properties vs network topology. ACS App. Mater. Interfaces.

[26] Krpovic S., Dam-Johansen K., Skov A.L. (2021). Importance of Mullins effect in commercial silicone elastomer formulations for soft robotics. J. Appl. Polym. Sci..

[27] Lee W.S., Yeo K.S., Andriyana A., Shee Y.G., Mahamd Adikan F.R. (2016). Effect of cyclic compression and curing agent concentration on the stabilization of mechanical properties of PDMS elastomer. Mater. Des..

[28] Clément F., Bokobza L., Monnerie L. (2001). On the Mullins effect in silica-filled polydimethylsiloxane networks. Rubber Chem. Tech..

[29] Ehrburger-Dolle F., Morfin I., Bley F., Livet F., Heinrich G., Richter S., Piché L., Sutton M. (2012). XPCS investigation of the dynamics of filler particles in stretched filled elastomers. Macromolecules.

[30] Ehrburger-Dolle F., Morfin I., Bley F., Livet F., Heinrich G., Piché L., Sutton M. (2014). Experimental clues of soft glassy rheology in strained filled elastomers. J. Polym. Sci. Part B Polym. Phys..

[31] Bergström J. (2015). Mechanics of Solid Polymers.

[32] Wang Z., Li S., Dongya W., Zhao J. (2015). Mechanical properties, Payne effect, and Mullins effect of thermoplastic vulcanizates based on high-impact polystyrene and styrene–butadiene rubber compatibilized by styrene–butadiene–styrene block copolymer. J. Thermopl. Compos. Mater..

[33] Boyce M.C., Socrate S., Kear K., Yeh O., Shaw K. (2001). Micromechanisms of deformation and recovery in thermoplastic vulcanizates. J. Mech. Phys. Sol..

[34] Boyce M.C., Yeh O., Socrate S., Kear K., Shaw K. (2001). Micromechanics of cyclic softening in thermoplastic vulcanizates. J. Mech. Phys. Sol..

[35] Marckmann G., Verron E., Gornet L., Chagnon G., Charrier P., Fort P. (2002). A theory of network alteration for the Mullins effect. J. Mech. Phys. Sol..

[36] Plagge J., Klüppel M. (2020). Micromechanics of stress-softening and hysteresis of filler reinforced elastomers with applications to thermo-oxidative aging. Polymer.

[37] Uthaipan N., Junhasavasdikul B., Nakason C., Thitithammawong A. (2015). Prediction models for the key mechanical properties of EPDM/PP blends as affected by processing parameters and their correlation with stress relaxation and phase morphologies. Polym. Adv. Tech..

[38] Andrady A.L., Llorente M.A., Mark J.E. (1980). Model networks of end-linked polydimethylsiloxane chains. VII. Networks designed to demonstrate non-Gaussian effects related to limited chain extensibility. J. Chem. Phys..

[39] Mihai L.A., Goriely A. (2017). How to characterize a nonlinear elastic material? A review on nonlinear constitutive parameters in isotropic finite elasticity. Proc. R. Soc. A Math. Phys. Eng. Sci..

